# Impact of Wildfire Smoke on Respiratory Disease Associated Healthcare Utilization in Gang‐Won Province, South Korea, in 2017

**DOI:** 10.1029/2025GH001332

**Published:** 2026-01-28

**Authors:** Min‐Taek Lee, Hoyoung Cha, Ju Won Lee, Jongjin Baik, Hae In Jung, Kyoung Min Moon, Changhyun Jun, Sun‐Young Jung, Kang‐Mo Gu

**Affiliations:** ^1^ College of Pharmacy Chung‐Ang University Seoul Korea; ^2^ Department of Global Innovative Drugs The Graduate School of Chung‐Ang University Seoul Korea; ^3^ Department of Civil, Environmental and Architectural Engineering Korea University Seoul Korea; ^4^ School of Civil, Environmental and Architectural Engineering Korea University Seoul Korea; ^5^ Department of Internal Medicine College of Medicine Chung‐Ang University Seoul South Korea; ^6^ Department of Internal Medicine Division of Pulmonary and Allergy Medicine Chung‐Ang University Hospital Seoul Korea; ^7^ Biomedical Research Institute Chung‐Ang University Hospital Seoul South Korea

**Keywords:** wildfire, healthcare utilization, air pollutants, wildfire smoke, respiratory diseases

## Abstract

This study aimed to elucidate the association between wildfire smoke exposure and healthcare utilization for respiratory diseases in Samcheok (City), Gangwon Province, South Korea, focusing on a major wildfire that occurred on 6–9 May 2017. The relative risks (RRs) of healthcare utilization for respiratory diseases in a direct‐exposure area (Samcheok) during (6–9 May 2017) and post (10 May–6 June 2017) wildfire periods, relative to the pre‐wildfire period (22 April–5 May 2017) were analyzed. The post‐wildfire period was divided into immediate and extended, each with a 2‐week interval. Additionally, the relative risk ratios (RRRs) of healthcare utilization were analyzed for the same period in 2018, when no wildfire occurred. In the direct‐exposure area (Samcheok), there were increased RRs of respiratory disease healthcare utilization for all ages in the wildfire (RR = 1.81, 95% confidence intervals [CI]: 1.67–1.96) and extended post‐wildfire (RR = 1.26, 95% CI: 1.20–1.33) periods. The highest risk was observed in children aged <9 years in the wildfire (RR = 2.20, 95% CI: 2.04–2.38) and extended post‐wildfire (RR = 1.44, 95% CI: 1.37–1.52) periods. Compared with that of the corresponding periods in 2018, significant increases in the RRRs were observed during the wildfire (RRR = 1.30, 95% CI: 1.15–1.45) and extended post‐wildfire (RRR = 1.75, 95% CI: 1.61–1.91) periods. The wildfire in Gangwon province significantly increased healthcare utilization for respiratory diseases during the wildfire and post‐wildfire periods.

## Plain Language Summary

A major wildfire occurred in Samcheok, South Korea, from 6 to 9 May 2017, leading to increased healthcare visits for respiratory problems. This study examined how wildfire smoke affected people's need for medical care in the city of Samcheok, where the fire occurred, and in Donghae, a nearby city. The research compared medical visits during the wildfire, immediately after, and weeks later to a period before the fire. The results showed a clear rise in hospital visits for breathing issues, especially among children under 9 years old, whose risks were highest during and after the fire. Even in Donghae, a city less directly affected, a small increase in respiratory healthcare visits was observed. By comparing these findings to the same time in 2018, when there was no wildfire, the study confirmed that wildfire smoke had both immediate and lingering impacts on health. This research highlights the serious health risks posed by wildfire smoke, particularly to vulnerable groups like children, and the need for strong public health measures during and after such events.

## Key Points

- Even short‐term exposure to small wildfires significantly increase the risk of healthcare utilization for respiratory diseases
- Exposure to wildfire smoke had immediate and delayed effects on the respiratory diseases healthcare utilization

## 1 Introduction

The frequency, intensity, and adverse effects of wildfires are increasing globally. Climate change, through global warming, contributes to the expansion of arid areas, causing an increased occurrence of large‐scale wildfires (Masson‐Delmotte et al., 2018; Sun et al., 2019; Wotton et al., 2017; Xu et al., 2020). Wildfire smoke‐generated air pollutants have various adverse effects on society, the economy, and public health worldwide (Cascio, 2018; Sun et al., 2019; Xu et al., 2020). The major air pollutants in wildfire smoke, including particulate matter (PM), carbon monoxide (CO), nitrogen oxides (NO_2_ and NO), ozone (O_3_), and volatile organic compounds, pose a critical threat to human health (Stone et al., 2019; Tao et al., 2020; Urbanski et al., 2008; Xu et al., 2020). PM is the primary pollutant emitted during wildfires, characterized by small size and high toxicity (Cascio, 2018; Dong et al., 2017; Makkonen et al., 2010; Verma et al., 2009). Even short‐term exposure to wildfire PM causes damage to the respiratory system and exacerbates respiratory diseases (J. C. Liu et al., 2017; Mahsin et al., 2022; Reid et al., 2019). Additionally, during wildfires, increased daily PM levels have been associated with an increased risk of mortality from any cause compared with those of the existing urban ambient air pollutants (Cascio, 2018; Reid, Brauer, et al., 2016; Xu et al., 2020). Similarly, the negative health impacts of short‐term exposure to wildfire CO (dos SANTOS et al., 2018) and O_3_ (Reid et al., 2019) have been reported. Wildfire smoke reportedly influences healthcare utilization significantly owing to the occurrence and exacerbation of respiratory disease, including chronic obstructive pulmonary disease (COPD), asthma, and pneumonia (Delfino et al., 2009; Hutchinson et al., 2018; J. C. Liu et al., 2015, 2017; Reid, Brauer, et al., 2016; Reid, Jerrett, et al., 2016; Reid & Maestas, 2019; Reid et al., 2019; Youssouf et al., 2014)

South Korea comprises over 63% mountainous terrains, and more than 400 wildfires, large and small, occur annually (Korea Forest Service, 2020). The Gangwon Province is in northeastern South Korea, and it is vulnerable to wildfires owing to its forest coverage of 81.5% (Korea Forest Service, 2020; Sim et al., 2020). The frequency and intensity of wildfires in South Korea could increase owing to global warming, necessitating an analysis of their impact on the public health of wildfire‐prone areas (Sun et al., 2019). While previous research has primarily focused on North America and Australia, studies on the health impacts of wildfires in South Korea remain limited. To address this gap, this study aimed to analyze the impacts of wildfires on healthcare utilization among the residents of wildfire‐exposed areas in Gangwon Province, South Korea. Wd utilized the National Health Insurance Service (NHIS) database allowing for a comprehensive analysis of long‐term healthcare utilization patterns. Furthermore, we utilized satellite data to effectively identify wildfire‐exposed areas, providing a valuable approach for real‐time monitoring and risk assessment. These findings can serve as a policy framework for optimizing healthcare resource allocation during future wildfire events.

## 2 Methods

### 2.1 Study Area and Period

In this study, a major wildfire that occurred in Samcheok (city) within Gangwon Province was selected. The Gangwon Province in the northeast of South Korea has a territory of 16,871.13 km^2^. The area comprises seven cities and 11 counties (Korean Statistical Information Service, 2024). The wildfire began on 6 May 2017, at 11:42 a.m. and was extinguished on 9 May 2017, at 11:20 a.m (Korea Forest Service, 2020). The wildfire in Samcheok destroyed 765 ha of forest, and it was the first to reach the “severe” level since the initiation of South Korea's wildfire alert system (Korea Forest Service, 2020). Additionally, it resulted in casualties (one death and four injuries) and property damage amounting to KRW 13.2 billion (Korea Forest Service, 2020).

**Figure 1:**
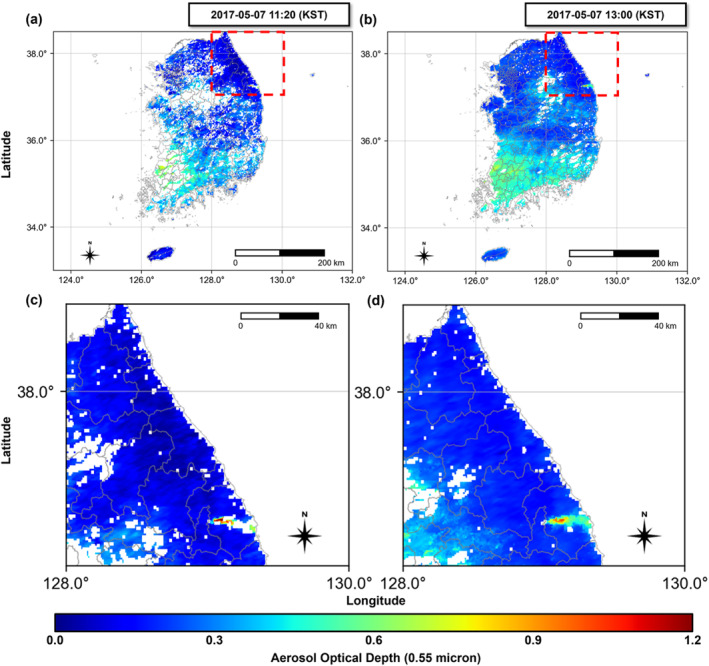
Aerosol optical depth (AOD) for measuring MCD19A2 Satellite Data: (a) 11:20 (KST) 7 May 2017, (b) 13:00 (KST) 7 May 2017. Spread of AOD at the specific location where the wildfire occurred: (c) 11:20 (KST) 7 May 2017, (d) 13:00 (KST) 7 May 2017 White areas represent regions with no detection.

**Figure 2:**
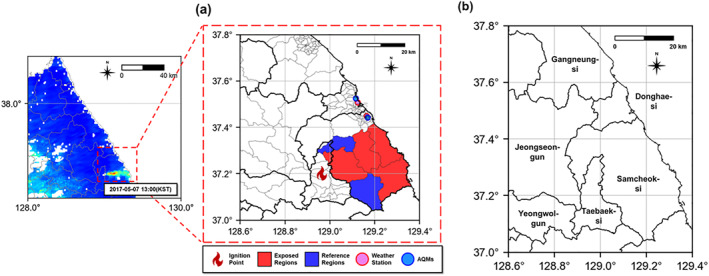
Wildfire‐exposed area (a) Locations of Ignition of Air Quality Monitoring systems, Weather Station, and Affected Areas on 6 May 2017 (b) Administrative Divisions of Gangwon‐do (Samcheok (Direct‐exposure area), Donghae (Indirect‐exposure area)).

The wildfire‐exposed areas were defined using satellite‐based data (MCD19A2 data from the Terra and Aqua satellites) by measuring the Aerosol Optical Depth (AOD) (Jang et al., 2019; Jia et al., 2020; D. S. Kim & Lee, 2016; Lee et al., 2017; Saim & Aly, 2024; Zhu et al., 2023). AOD is commonly used to quantify air pollution, as it aids in determining the reduction in the sunbeam intensity caused by dust and haze from wildfire smoke (Li et al., 2010; Wei et al., 2020; Xia et al., 2007). AOD variability was evident on 7 May 2017, in Gangwon Province (Figure 1). Additionally, we used Geographic Information Systems to visualize wildfire‐exposed areas at the township level and identified the locations of Air Quality Monitoring systems (AQMs) within the regions initially selected using satellite data (Figure 2). Based on this data, we defined Samcheok as a direct‐exposure area, where the ignition points and AOD changes were directly observed, and Donghae (city) as an indirect‐exposure area.

The wildfire period was defined as the duration between wildfire ignition and containment (6–9 May 2017). The periods before and after the wildfire were defined at 2‐week intervals as pre‐wildfire (22 April–5 May 2017), immediate post‐wildfire (10–23 May 2017), and extended post‐wildfire (24 May–6 June 2017) periods. As a control, data from the same periods in the year after the wildfire (2018) were compiled.

### 2.2 Air Pollutant Data

The air pollutant data analyzed in this study included PM (PM_2.5_ and PM_10_, μg/m^3^), O_3_ (ppm), NO_2_ (ppm), CO (ppm), and SO_2_ (ppm). The air pollutant concentrations during the study period were obtained from AQMs in Samcheok and Donghae, with the data provided by Air Korea of the Korea Environment Corporation. The hourly concentrations of the air pollution indicators were calculated as daily averages, which were subsequently set as representative values for the daily data. The hourly wind direction (16 compass points) and relative humidity (%) were measured using weather observation data from individual stations provided by the Korea Meteorological Administration's Open MET Data Portal (Korea Meteorological Administration, 2024).

To objectify the changes in air pollutant concentrations in wildfire‐exposed areas, normalization was performed. Over 6 years (2015–2020), the daily average concentration of each air pollution indicator was calculated. Maximum‐minimum normalization was performed using the calculated maximum and minimum values (Equation 1).

(1) $$\begin{array}{c} X_{\text{scaled}}\left(\%\right)=\frac{X_{\text{Day}}-X_{\min}}{X_{\max}-X_{\min}}\times 100\left(\%\right) \end{array}$$

In Equation 1, $X_{\text{Day}}$ represents the daily average hourly air pollutant concentrations, and $X_{\max}$ and $X_{\min}$ represent the maximum and minimum air pollutant concentrations between 2015 and 2020, respectively. The calculated $X_{\text{scaled}}$ represents the normalized air pollutant concentrations for each monitoring station and air pollutant indicator. The converted air pollution values, which ranged from 0 to 1, were multiplied by 100 to convert them into percentages. Air pollutant and weather data processing were performed using Python (v3.10).

### 2.3 Healthcare Utilization Data

We used the data from the National Health Insurance Service (NHIS) ‐ National Health Information Database (NHIS‐2023‐1‐284), which contains reimbursement claims from healthcare facilities for over 50 million individuals in South Korea. Diagnoses were coded using the International Classification of Diseases, 10th Revision. Healthcare utilization was aggregated using outpatient department visits, emergency department visits, and hospitalizations for respiratory disease according to study periods. The date of the first healthcare utilization was included if the patient had more than two visits in each period. The healthcare utilization data was provided at the city level.

### 2.4 Covariates

Demographic information, including age, sex, comorbidities, and income level, during the wildfire period (6–9 May 2017), was obtained. The respiratory diseases included in this study were pneumonia (J13 and J15‐18), acute bronchitis (J20), acute bronchiolitis (J21), unspecified lower respiratory tract infection (J22), COPD (J40‐44), asthma (J45‐46), and bronchiectasis (J47). Comorbidities were defined as claim codes within 1 year of the first date of wildfire occurrence. The comorbidities included interstitial lung diseases (J84), hypertensive disease (I10‐I15), dyslipidemia (E78), diabetes mellitus (E10‐E14), ischemic heart disease (I20‐I25), arrhythmias (I47‐I49), heart failure (I50), renal failure (N17‐N19), mental and behavioral disorders (F00–F99), lung cancer (C34), and cerebral infarction (I63).

### 2.5 Statistical Analysis

The baseline characteristics of the exposed area populations were presented as mean (standard deviation) for continuous variables or number (percentage) for categorical variables. To assess the direction of changes in pollutant concentrations between the pre‐wildfire and wildfire periods, the Mann–Kendall trend test was used. The resulting Slope‐values indicate whether a statistically significant monotonic trend existed: Slope >0 denotes an increasing trend, Slope <0 denotes a decreasing trend, and Slope = 0 indicates no apparent trend. *P*‐values less than 0.05 were considered statistically significant. Healthcare utilization was estimated according to age groups, and the incidence rates were calculated by dividing the number of healthcare users by the annual population for each area. Age groups were classified according to analytical purpose: 10‐year intervals (0–9, 10–19, 20–29, etc.) were used for descriptive analyses, while participants were categorized as <10, 10–19, 20–64, and ≥65 years for risk analyses based on standard epidemiologic and clinical definitions. We used a quasi‐Poisson regression model with a log‐link function to enable overdispersion and estimated the association between daily healthcare utilization and air pollution indicators (Bhaskaran et al., 2013; Mahsin et al., 2022). The statistical model was adjusted for weekend or holiday days, wind direction, and relative humidity. The relative risks (RRs) were calculated for all‐cause or disease‐specific medical utilization caused by air pollution from wildfires, using the period before the wildfires as the reference. RRs were evaluated according to exposure to wildfire on the day of healthcare utilization (Lag0), the day before the healthcare utilization (Lag1), up to 3 days before the healthcare utilization (Lag3), and a 3‐day average encompassing Lag0, Lag1, and Lag2 (Mahsin et al., 2022). Among the five lag models, the best‐fit model was selected based on the Akaike Information Criterion (AIC), Corrected Akaike Information Criterion (AICC), and Bayesian Information Criterion values (BIC) (Wettstein et al., 2018). The model fitting test indicated that the 3‐day average lag model had the lowest generalized cross‐validation value, making it the most suitable model (Table S1 in Supporting Information S1). We estimated confidence intervals (CI) for RRs using 200 bootstrap samples with replacement (Schwarz et al., 2023; Su et al., 2015). Additionally, we calculated the ratio of relative risks (RRRs) by comparing the 2017 RRs estimated with those of 2018 over the same periods to assess the excess risk attributable to wildfires in 2017 (Altman & Bland, 2003). All analysis were performed using SAS Enterprise Guild software (version 7.1; SAS Institute, Cary, NC, USA). This study protocol was approved by the Chung‐Ang University Hospital Institutional Review Board (IRB) (IRB number 2207‐019‐19428). The requirement for informed consent was waived because all participants were de‐identified.

## 3 Results

**Figure 3:**
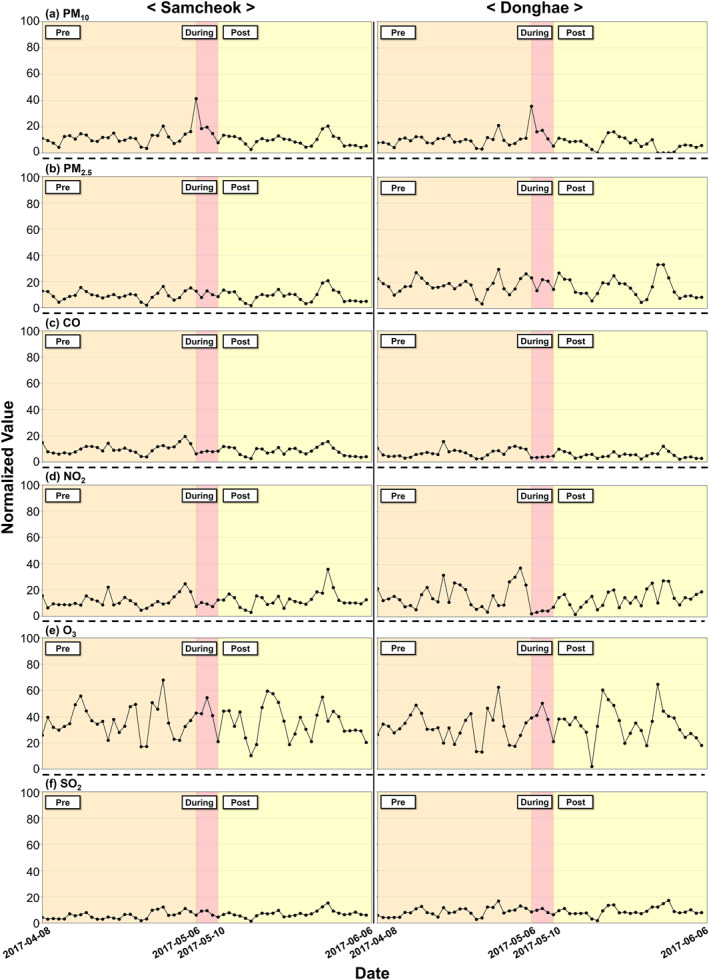
Distribution of normalized mean concentrations of air pollutants by wildfire‐exposed areas. [(a) PM_10_ (b) PM_2.5_ (c) CO (e) O_3_ (d) NO_2_ (f) SO_2_].

Table 1 presents the normalized values of the daily average concentrations of air pollutants in direct‐exposure area (Samcheok). Among the air pollutants, according to the Mann–Kendall trend test, PM_10_ exhibited a notable increasing trend, with a calculated slope of 0.11 (*p* < 0.05). Furthermore, increasing trends were also observed in O_3_, PM_2.5_, and SO_3_ concentrations. The daily normalized concentration of PM_10_ showed a notable increasing trend during the wildfire period, and similar trends were observed in indirect‐exposure area (Figure 3).

**Table 1:** Baseline and Normalized Concentration Values of Air Pollutants by Period in Direct‐Exposure Area (Samcheok)

	Variable	Pre‐wildfire period		Wildfire period		Trend until wildfire period	Immediate post‐wildfire period		Extended post‐wildfire period	
		Min‐Max	Mean (SD)	Min‐Max	Mean (SD)	Slope (*P*‐value*)	Min‐Max	Mean (SD)	Min‐Max	Mean (SD)
B	PM_2.5_ (μg/m^3^)	4.0–54.0	22.27 (9.37)	10.0–63.0	24.23 (12.39)	1.79 × 10^−2^ *	8.0–53.0	22.61 (8.13)	4.0–68.0	19.77 (13.15)
	PM_10_ (μg/m^3^)	15.0–130.0	53.54 (21.74)	31.0–323.0	105.73 (64.83)	1.10 × 10^−1^ *	4.0–99.0	48.87 (14.6)	6.0–142.0	39.65 (25.26)
	O_3_ (ppb)	2.0–103.0	51.93 (23.4)	20.0–88.0	59.92 (13.15)	2.33 × 10^−5^ *	1.0–113.0	53.38 (24.6)	2.0–96.0	44.77 (20.56)
	NO_2_ (ppb)	3.0–55.0	13.76 (8.85)	3.0–30.0	8.97 (4.58)	No trend	3.0–39.0	12.52 (7.15)	4.0–77.0	14.96 (11.86)
	CO (ppb)	200.0–1900.0	410.32 (192.33)	200.0–600.0	267.37 (86.38)	No trend	100.0–700.0	329.04 (116.1)	100.0–800.0	289.25 (157.42)
	SO_2_ (ppb)	1.0–8.0	2.68 (1.36)	1.0–5.0	2.75 (0.95)	2.75 × 10^−6^ *	1.0–6.0	2.34 (0.77)	1.0–11.0	2.92 (1.44)
	WD (deg)	1.9–358.6	183.96 (84.66)	9.9–358.0	179.45 (107.21)	No trend	1.5–359.7	180.43 (88.38)	0.1–359.8	170.6 (103.82)
	RH (%)	11.0–93.0	44.07 (18.65)	16.0–97.0	60.19 (27.39)	2.86 × 10^−2^ *	18.0–96.0	55.47 (22.81)	25.0–95.0	62.96 (18.07)
N	PM_2.5_	1.79–24.11	9.94 (4.18)	4.46–28.12	10.82 (5.53)	2.83 × 10^−2^ *	3.57–23.66	10.09 (3.63)	1.79–30.36	8.82 (5.87)
	PM_10_	3.36–29.15	12.0 (4.87)	6.95–72.42	23.71 (14.54)	3.41 × 10^−2^ *	0.9–22.2	10.96 (3.27)	1.35–31.84	8.89 (5.66)
	O_3_	1.5–77.44	39.04 (17.59)	15.04–66.17	45.05 (9.89)	2.26 × 10^−2^ *	0.75–84.96	40.13 (18.49)	1.5–72.18	33.66 (15.45)
	NO_2_	2.88–52.88	13.24 (8.51)	2.88–28.85	8.62 (4.4)	No trend	2.88–37.5	12.04 (6.88)	3.85–74.04	14.38 (11.41)
	CO	5.56–52.78	11.4 (5.34)	5.56–16.67	7.43 (2.4)	No trend	2.78–19.44	9.14 (3.23)	2.78–22.22	8.03 (4.37)
	SO_2_	2.78–22.22	7.44 (3.78)	2.78–13.89	7.63 (2.64)	3.43 × 10^−2^ *	2.78–16.67	6.49 (2.15)	2.78–30.56	8.12 (3.99)
	WD	0.53–99.61	51.1 (23.52)	2.75–99.44	49.85 (29.78)	No trend	0.42–99.92	50.12 (24.55)	0.03–99.94	47.39 (28.84)
	RH	9.18–92.86	42.92 (19.03)	14.29–96.94	59.38 (27.95)	3.32 × 10^−2^ *	16.33–95.92	54.56 (23.28)	23.47–94.9	62.21 (18.44)

*Note*. B = baseline concentration, N = normalized concentration, SD = Standard deviation, WD = Wind direction, RH = Relative humidity. The Slope‐value was calculated using the Mann–Kendall trend test to assess the direction of changes in pollutant concentrations between Pre‐wildfire period and Wildfire period. The resulting Slope‐values indicate whether a statistically significant monotonic trend existed: Slope >0 denotes an increasing trend. Statistical significance was denoted as * for *P* < 0.05, while “No trend” was indicated when no statistically significant trend was observed. Pre‐wildfire period (22 April 2017–5 May 2017), Wildfire period (6 May 2017–9 May 2017), Immediate post‐wildfire period (10 May 2017–23 May 2017), and Extended post‐wildfire period (24 May 2017–6 June 2017).

As of May 2017, the populations of Samcheok (the direct‐exposure area) and Donghae (the indirect‐exposure area) were 69,212 and 92,814, respectively. The number of healthcare utilization cases was 18,742 in Samcheok and 27,200 in Donghae. The mean age of the healthcare utilization patients in the direct‐exposure areas was 45.8 years, with a higher proportion of women (55.9%). During the study period, medical utilization for acute bronchitis was the highest (2,553 cases), followed by chronic respiratory diseases, such as COPD and asthma. There were no significant differences in age or sex between the direct (Samcheok) and indirect (Donghae) exposure areas. Several baseline characteristics showed non‐uniform distributions between the two regions (Table 2).

**Table 2:** Baseline Characteristics of the Population Utilizing Healthcare Services in Wildfire‐Exposed Areas

	Samcheok (Direct‐exposure area)	Donghae (Indirect‐exposure area)	*p*‐value
Total cases	18,742	27,200	
Age			0.231
Mean (SD)	45.8 (24.6)	42.4 (24.2)	
Median (Q1‐Q3)	50 (23–65)	45 (20–62)	
0–9 years	1,933 (10.3)	3,436 (12.6)	
10–19 years	1,814 (9.7)	3,254 (12.0)	
20–29 years	1,805 (9.6)	2,169 (8.0)	
30–39 years	1,674 (8.9)	2,861 (10.5)	
40–49 years	2,105 (11.2)	3,554 (13.1)	
50–59 years	2,905 (15.5)	4,119 (15.1)	
60–69 years	2,904 (15.5)	3,799 (14.0)	
70–79 years	2,338 (12.5)	2,762 (10.2)	
≥80 years	1,264 (6.7)	1,246 (4.6)	
Sex (%)			0.8431
Male	8,260 (44.1)	12,013 (44.2)	
Female	10,482 (55.9)	15,187 (55.8)	
Respiratory disease			
Pneumonia (J13‐18, except J14)	203 (1.1)	216 (0.8)	0.0014
Acute bronchitis (J20)	2,553 (13.6)	4,478 (16.5)	<0.0001
Acute bronchiolitis (J21)	73 (0.4)	94 (0.4)	0.4421
Unspecified lower respiratory tract infection (J22)	72 (0.4)	847 (3.1)	<0.0001
COPD (J40‐44)	1,007 (5.4)	1,128 (4.2)	<0.0001
Asthma (J45‐46)	1,095 (5.8)	889 (3.3)	<0.0001
Bronchiectasis (J47)	39 (0.2)	35 (0.1)	0.0370
Income level			<0.0001
0‐5	4,385 (23.4)	5,641 (20.7)	
6–11	4,289 (22.9)	6,344 (23.3)	
12–15	3,677 (19.6)	5,056 (18.6)	
16‐20	5,774 (30.8)	7,922 (29.1)	
missing	617 (3.3)	2,237 (8.2)	
Comorbidity			
Interstitial lung diseases (J84)	60 (0.3)	28 (0.1)	<0.0001
Hypertensive disease (I10‐I15)	5,789 (30.9)	7,513 (27.6)	<0.0001
Dyslipidemia (E78)	4,869 (26.0)	6,662 (24.5)	0.0003
Diabetes mellitus (E10‐E14)	2,938 (15.7)	3,787 (13.9)	<0.0001
Ischemic heart disease (I20‐I25)	1,309 (7.0)	1,437 (5.3)	<0.0001
Arrhythmias (I47‐I49)	404 (2.2)	693 (2.6)	0.0068
Heart failure (I50)	1,395 (7.4)	881 (3.2)	<0.0001
Renal failure (N17‐N19)	245 (1.3)	323 (1.2)	0.2541
Mental and Behavioral Disorders (F00–F99)	4,135 (22.1)	5,616 (20.7)	<0.0001
Lung cancer (C34)	72 (0.4)	106 (0.4)	0.9251
Cerebral infarction (I63)	490 (2.6)	648 (2.4)	0.1157

*Note*. SD = Standard deviation, COPD = Chronic obstructive pulmonary disease. Data are presented as number (%), mean (standard deviation), or median (interquartile range).

Table 3 presents the frequency and incidence of respiratory disease healthcare utilization in the population of the wildfire‐exposed areas by age group. The incidence of healthcare utilization was highest during the wildfire period, with 7,390 patients utilizing medical services (3,038 in Samcheok and 4,352 in Donghae), and a decreasing trend in incidence was observed in the post‐wildfire period. Similar trends were observed in the direct and indirect‐exposure areas (Table 3). Outpatient clinic visits were the most common form of medical utilization (Table S2 in Supporting Information S1).

**Table 3:** Frequency and Incidence of Respiratory Disease Healthcare Utilization Among the Population of Wildfire‐Exposed Areas by Age Groups

Age	Pre‐wildfire period				Wildfire period			Immediate post‐wildfire period			Extended post‐wildfire period		
Groups (years)	Pop*	Count	PY	IR	Count	PY	IR	Count	PY	IR	Count	PY	IR
(a) Samcheok (Direct‐exposure area)													
All ages	69,212	8,341	968,968	0.009	3,038	276,848	0.011	8,593	968,968	0.009	8,180	968,968	0.008
0–9	4,459	899	62,426	0.014	347	17,836	0.019	842	62,426	0.013	870	62,426	0.014
10–19	6,910	494	96,740	0.005	151	27,640	0.005	431	96,740	0.004	407	96,740	0.004
20–29	8,477	499	118,678	0.004	180	33,908	0.005	488	118,678	0.004	411	118,678	0.003
30–39	6,888	547	96,432	0.006	200	27,552	0.007	592	96,432	0.006	518	96,432	0.005
40–49	10,102	723	141,428	0.005	251	40,408	0.006	787	141,428	0.006	730	141,428	0.005
50–59	12,062	1,300	168,868	0.008	459	48,248	0.010	1,374	168,868	0.008	1,281	168,868	0.008
60–69	9,527	1,539	133,378	0.012	579	38,108	0.015	1,681	133,378	0.013	1,582	133,378	0.012
70–79	7,013	1,520	98,182	0.015	602	28,052	0.021	1,580	98,182	0.016	1,567	98,182	0.016
≥80	3,297	820	46,158	0.018	269	13,188	0.020	818	46,158	0.018	814	46,158	0.018
(b) Donghae (Indirect‐exposure area)													
All ages	92,814	11,714	1,299,396	0.009	4,352	371,256	0.012	12,040	1,299,396	0.009	11,290	1,299,396	0.009
0–9	7,838	1,608	109,732	0.015	599	31,352	0.019	1,475	109,732	0.013	1,581	109,732	0.014
10–19	10,177	866	142,478	0.006	263	40,708	0.006	833	142,478	0.006	821	142,478	0.006
20–29	9,572	565	134,008	0.004	197	38,288	0.005	592	134,008	0.004	526	134,008	0.004
30–39	11,302	949	158,228	0.006	322	45,208	0.007	944	158,228	0.006	868	158,228	0.005
40–49	15,843	1,335	221,802	0.006	469	63,372	0.007	1,336	221,802	0.006	1,170	221,802	0.005
50–59	15,835	1,831	221,690	0.008	675	63,340	0.011	1,939	221,690	0.009	1,787	221,690	0.008
60–69	11,446	2,013	160,244	0.013	760	45,784	0.017	2,213	160,244	0.014	1,964	160,244	0.012
70–79	7,466	1,769	104,524	0.017	756	29,864	0.025	1,881	104,524	0.018	1,789	104,524	0.017
≥80	2,929	778	41,006	0.019	311	11,716	0.027	827	41,006	0.020	784	41,006	0.019

*Note*. Pop = total population of wildfire‐exposed areas; PY = person‐years; IR = incidence rate. Pre‐wildfire (22 April 2017–5 May 2017), wildfire (6 May 2017–9 May 2017), immediate post‐wildfire (10 May 2017–23 May 2017), and extended post‐wildfire (24 May 2017–6 June 2017) periods.

**Figure 4:**
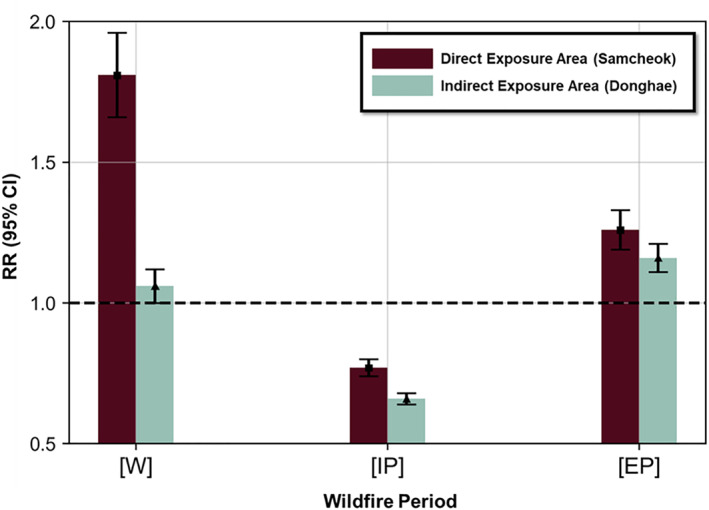
Relative Risk of Respiratory Disease Healthcare Utilization among the Populations of Wildfire‐Exposed Areas. [W] = wildfire period (6 May 2017–9 May 2017); [IP] = immediate post‐wildfire period (10 May 2017–23 May 2017); [EP] = extended post‐wildfire period (24 May 2017–6 June 2017). RR—Risk ratio, CI—Confidence interval.

Table 4 presents the RR and RRR analyses of all‐cause respiratory disease healthcare utilization in wildfire‐exposed areas during the wildfire and post‐wildfire periods. In both areas, an increased RR of healthcare utilization was observed during the wildfire and extended post‐wildfire periods (Figure 4).

**Table 4:** Relative Risks of Respiratory Disease Healthcare Utilization Among the Population of Wildfire‐Exposed Areas by Age Groups

Variable	Age group (year)	2017 [W] RR (95% CI)	2017 [IP] RR (95% CI)	2017 [EP] RR (95% CI)	2018 [W] RR (95% CI)	2018 [IP] RR (95% CI)	2018 [EP] RR (95% CI)	2017/2018 [W] RRR (95% CI)	2017/2018 [IP] RRR (95% CI)	2017/2018 [EP] RRR (95% CI)
(a) Samcheok (Direct‐exposure area)										
All	All age	1.81 (1.67–1.96)	0.77 (0.75–0.80)	1.26 (1.20–1.33)	1.40 (1.28–1.52)	0.79 (0.75–0.84)	0.72 (0.67–0.77)	1.30 (1.15–1.45)	0.97 (0.91–1.04)	1.75 (1.61–1.91)
	≥20	1.83 (1.68–1.98)	0.76 (0.74–0.79)	1.23 (1.17–1.29)	1.23 (1.13–1.33)	0.73 (0.69–0.77)	0.67 (0.62–0.71)	1.49 (1.32–1.67)	1.04 (0.97–1.11)	1.84 (1.69–2.00)
	0–9	2.20 (2.04–2.38)	0.83 (0.80–0.86)	1.44 (1.37–1.52)	1.61 (1.48–1.75)	0.88 (0.83–0.94)	0.79 (0.73–0.85)	1.37 (1.22–1.54)	0.94 (0.87–1.00)	1.83 (1.67–2.01)
	10–19	1.23 (1.13–1.33)	0.71 (0.69–0.74)	1.09 (1.03–1.15)	0.88 (0.82–0.96)	0.60 (0.56–0.63)	0.54 (0.51–0.58)	1.39 (1.24–1.55)	1.20 (1.12–1.28)	1.99 (1.83–2.17)
	20–65	1.90 (1.76–2.06)	0.80 (0.77–0.82)	1.19 (1.13–1.25)	1.32 (1.22–1.43)	0.82 (0.77–0.86)	0.69 (0.65–0.74)	1.44 (1.29–1.61)	0.98 (0.92–1.04)	1.72 (1.58–1.86)
	>65	1.76 (1.61–1.93)	0.71 (0.68–0.73)	1.24 (1.17–1.31)	1.45 (1.31–1.59)	0.75 (0.70–0.80)	0.74 (0.69–0.80)	1.22 (1.07–1.39)	0.94 (0.87–1.01)	1.67 (1.52–1.83)
(b) Donghae (Indirect‐exposure area)										
All	All age	1.06 (1.01–1.12)	0.66 (0.64–0.68)	1.16 (1.11–1.21)	2.21 (2.10–2.32)	1.30 (1.25–1.35)	1.42 (1.36–1.48)	0.48 (0.45–0.52)	0.51 (0.48–0.53)	0.81 (0.77–0.86)
	≥20	1.21 (1.14–1.27)	0.64 (0.62–0.66)	1.08 (1.03–1.12)	2.26 (2.14–2.37)	1.34 (1.29–1.40)	1.46 (1.40–1.52)	0.53 (0.50–0.58)	0.48 (0.45–0.50)	0.74 (0.70–0.78)
	0–9	0.71 (0.67–0.75)	0.71 (0.69–0.74)	1.57 (1.51–1.64)	2.31 (2.20–2.43)	1.29 (1.25–1.35)	1.44 (1.38–1.50)	0.31 (0.29–0.33)	0.55 (0.52–0.58)	1.09 (1.03–1.16)
	10–19	1.08 (1.02–1.13)	0.66 (0.64–0.68)	0.96 (0.92–1.00)	1.44 (1.37–1.51)	1.12 (1.07–1.16)	1.06 (1.02–1.10)	0.75 (0.70–0.80)	0.59 (0.57–0.62)	0.90 (0.85–0.96)
	20–65	1.13 (1.08–1.19)	0.67 (0.66–0.69)	1.07 (1.03–1.11)	2.07 (1.97–2.17)	1.28 (1.23–1.33)	1.34 (1.28–1.39)	0.55 (0.51–0.59)	0.53 (0.50–0.55)	0.80 (0.76–0.85)
	>65	1.40 (1.31–1.49)	0.59 (0.57–0.61)	1.07 (1.02–1.12)	2.66 (2.52–2.82)	1.40 (1.34–1.46)	1.69 (1.62–1.77)	0.52 (0.48–0.57)	0.42 (0.40–0.44)	0.63 (0.59–0.68)

*Note*. Reference—Pre‐wildfire period (April 22–May 5). [W] = wildfire period (May 6–May 9); [IP] = immediate post‐wildfire period (May 10–May 23); [EP] = extended post‐wildfire period (May 24–June 6). RR—Risk ratio, RRR—Ratio of RR, CI—Confidence interval. Quasi‐Poisson regression model with a log‐link function to allow for over‐dispersion to estimate the association of daily healthcare use with air pollution indicators, including PM, O_3_, NO_2_, CO, and SO_2_. The statistical model was adjusted for day of the weekend or holiday, wind direction, and relative humidity.

During the wildfire period, for all ages, the direct‐exposure area showed an 81% increase in the risk of healthcare utilization (RR = 1.81, 95% CI 1.67–1.96), which was higher than that in the indirect‐exposure area (RR = 1.06, 95% CI: 1.01–1.12). During the extended post‐wildfire period, the direct‐exposure area showed a 26% increase in the risk of healthcare utilization (RR = 1.26, 95% CI: 1.20–1.33), which was higher than that in the indirect‐exposure area (RR = 1.16, 95% CI: 1.11–1.21). The highest risk was observed in children aged <9 years (RR = 2.20, 95% CI: 2.04–2.38) in the direct‐exposure area during the wildfire period. In the direct‐exposure area in 2018, the RR of healthcare utilization increased during the wildfire period (May 6–9) with an RR of 1.40 (95% CI: 1.28–1.52) and decreased in the extended post‐wildfire period (RR = 0.72, 95% CI: 0.67–0.77). In contrast, during the wildfire period in 2017, the RR was significantly higher than that of 2018 (RR [95% CI] 1.81 [1.67–1.96] vs. 1.40 [1.28–1.52]), and a notable increase in RR was observed during the extended post‐wildfire period. In the direct‐exposure area, the RRRs were increased in the wildfire (RRR = 1.30, 95% CI: 1.15–1.45) and extended post‐wildfire (RRR = 1.75, 95% CI: 1.61–1.91) periods. In the indirect‐exposure area, a significant increase in healthcare utilization was observed during the same period in 2018 that experienced no wildfire.

For disease‐specific respiratory healthcare utilization, the risk of the studied respiratory diseases was significantly increased during the wildfire and extended post‐wildfire periods. This trend was more pronounced in adults aged ≥20 years. In the direct‐exposure area, children aged <9 years showed significant increases in RRs for pneumonia (RR = 2.48, 95% CI: 2.28–2.70), acute bronchitis (RR = 2.21, 95% CI: 2.04–2.38), and asthma (RR = 2.26, 95% CI: 2.07–2.46) compared with those in other age groups during the wildfire period. During the wildfire period, the risk of healthcare utilization for asthma (RR [95% CI] 1.81 [1.65–1.99] vs. 1.73 [1.60–1.88]) was significantly increased among the older population aged ≥65 years compared with those in younger adults. During the extended post‐wildfire period, the RRs for pneumonia (RR = 1.39, 95% CI: 1.31–1.47), acute bronchitis (RR = 1.44, 95% CI: 1.37–1.52), and asthma (RR = 1.52, 95% CI: 1.43–1.61) were notably higher in children aged <9 years than in other age groups. A similar trend was observed for the indirect‐exposure area. Consistent with the results for all‐cause respiratory healthcare utilization, the disease‐specific RRRs showed a more significant increase only in the direct‐exposure areas (Table S3 in Supporting Information S1).

## 4 Discussion

To our knowledge, this study is the first to show that wildfires in South Korea significantly increase healthcare utilization for respiratory diseases. We found that exposure to wildfire smoke had immediate and delayed effects on the respiratory diseases associated with healthcare utilization in children aged <9 years and older adults. Disease‐specific analysis further confirmed the worsening of respiratory conditions owing to wildfire smoke exposure. In 2018, the direct‐exposure area showed increased RR of healthcare utilization during the wildfire period (May 6–9) but not in the post‐wildfire period. In contrast, during the wildfire period in 2017, the RR was significantly higher than that in 2018, and a notable increase in RR was observed during the extended post‐wildfire period. This finding emphasizes that exposure to wildfire smoke significantly increases the risk of healthcare utilization in direct‐exposure areas.

This study demonstrates the utility of satellite data in identifying wildfire‐exposed areas, enabling targeted interventions and optimized healthcare resource allocation during wildfire events. By detecting increases in PM concentrations by wildfire smoke utilizing satellite data, authorities can enhance response capacity at healthcare facilities of wildfire‐prone area and implement early interventions such as mask distribution and evacuation shelters for vulnerable populations, including children and older adults. While South Korea has some public health measures in place, such as disaster alerts and air quality advisories, systematic interventions like protective mask distribution, temporary evacuation programs, and improved healthcare access remain insufficient. And for the delayed healthcare utilization patterns of extended post‐wildfire period, strengthening public health strategies is essential. Proactive measures—including expanding healthcare access, raising public awareness, and implementing targeted interventions—should be prioritized to mitigate the delayed health effects of exposure to wildfire smoke. Additionally, integrating real‐time satellite data with early warning systems could facilitate rapid response efforts and ensure efficient resource allocation for affected populations.

Exposure to wildfire smoke reportedly increases the incidence and exacerbation of respiratory diseases and the associated healthcare utilization (Black et al., 2017; Cascio, 2018; Reid, Brauer, et al., 2016). Mahsin et al. demonstrated that short‐term exposure to wildfire‐related PM_2.5_ significantly increased healthcare utilization during wildfire and post‐wildfire periods, particularly among children and individuals in Canada (Mahsin et al., 2022). Aguilera et al. found that wildfire‐specific PM_2.5_ caused a significant increase in respiratory hospitalizations compared with those of non‐wildfire PM_2.5_ in California, US. (Aguilera et al., 2021). We confirmed that the risk of respiratory healthcare utilization owing to exposure to wildfire smoke was increased in disease‐specific analyses. Studies have confirmed that the risk of medical utilization is increased for respiratory infections, such as pneumonia (Aguilera et al., 2021; Gan et al., 2017; Hutchinson et al., 2018) and airway diseases (COPD (Alman et al., 2016; Gan et al., 2017; Reid, Jerrett, et al., 2016) and asthma (Alman et al., 2016; Gan et al., 2017; Haikerwal et al., 2016; Hutchinson et al., 2018; Reid, Jerrett, et al., 2016)) by exposure to wildfire smoke. Wildfire smoke reportedly exerts greater toxic effects than ambient air pollutants do because it markedly exacerbates the induction of oxidative stress, cell death, and inflammation (Y. H. Kim et al., 2018; Wegesser et al., 2009; Williams et al., 2013). Additionally, it alters the macrophages and human lung epithelial cells, inducing the onset or exacerbation of respiratory infections and airway diseases such as asthma and COPD (Adetona et al., 2016; Nakayama Wong et al., 2011; Roscioli et al., 2018). Asthma is among the most significantly affected conditions by wildfires (Borchers Arriagada et al., 2019; Reid & Maestas, 2019; Verma et al., 2009). In our study, we observed a marked increase in asthma‐related medical utilization during the wildfire and extended post‐wildfire periods, particularly in children and adolescents. Given that asthma occurs and persists across all age groups, the finding that wildfires in Korea influence healthcare utilization among patients with asthma is meaningful.

A significant strength of this study is the analysis of the post‐wildfire periods, which were divided into two, during which a significant increase in healthcare utilization for respiratory diseases was observed in the extended post‐wildfire period in the direct‐exposure areas. In this study, PM_10_, PM_2.5_, and O_3_ levels increased during the wildfire period and decreased subsequently, suggesting that exposure to these factors could have a delayed impact on respiratory diseases. Studies have indicated that wildfire PM has a delayed impact on the occurrence of respiratory diseases post‐wildfire period (Aguilera et al., 2021; Landguth et al., 2020; Mahsin et al., 2022). PM_2.5_ from recent wildfires in the mountainous regions of the western United States reportedly caused increased influenza cases 1 month lag periods (Landguth et al., 2020) In our study, there was a significant increase in the RR of healthcare utilization among children aged <9 years during the wildfire and extended post‐wildfire periods, with this trend being particularly pronounced for pneumonia, acute bronchitis, and asthma. Similarly, studies have confirmed that exposure to wildfire smoke could adversely affect the respiratory health of children and adolescents during the post‐wildfire periods (Horne et al., 2018; Mahsin et al., 2022; Zhang et al., 2024). There are multiple causes of this delayed reaction. Wildfire smoke‐related inflammation intensifies over time, leading to delayed exacerbation of pre‐existing chronic respiratory diseases, such as asthma and COPD, and worsened symptoms after days or even weeks (Reid, Brauer, et al., 2016; Xu et al., 2020; Zhang et al., 2024). Additionally, the toxic components of wildfire smoke weaken the immune system, increasing susceptibility to infections and contributing to delayed healthcare utilization, as respiratory infections or other health complications emerge (Landguth et al., 2020; Zhang et al., 2024). Moreover, wildfire smoke could enhance allergic sensitization to other allergens or elicit a delayed immune response (Blando et al., 2022; Bowman et al., 2024). This indicates that children require more awareness during wildfires and in the subsequent weeks, particularly after 3 weeks, because there is a higher likelihood of respiratory complications occurring and worsening.

Furthermore, we identified an increased risk of healthcare utilization by asthma, particularly among persons aged ≥65 years. Our results confirm that older patients aged ≥65 years are particularly vulnerable to the exacerbation of respiratory diseases owing to wildfire smoke exposure (Borchers Arriagada et al., 2019; Kondo et al., 2019). The vulnerability of older patients to exposure to wildfire smoke is caused by the usual pre‐existing respiratory and cardiovascular diseases among this population (J. C. Liu et al., 2015; Reid & Maestas, 2019; Xu et al., 2020). Additionally, owing to the aging lung process, the lung defense mechanisms in older patients are weakened; thus, exposure to toxic substances, such as wildfire smoke, more likely increases the risk of respiratory diseases in this population (Lowery et al., 2013; Schneider et al., 2021).

In this study, changes in air pollutants owing to wildfire smoke were assessed using the daily concentrations at observation stations in the exposed areas. To accurately demonstrate the impact of wildfires, we normalized the mean concentration of each air pollutant based on the data from the 6 years before and after the wildfire period. This approach enhanced the objectivity of our analysis by improving the understanding of how wildfire‐induced air pollutants affect healthcare utilization. Studies have reported that wildfire‐induced air pollution primarily affects public health through changes in the PM, CO, and O_3_ levels (Reid & Maestas, 2019; Tao et al., 2020; Urbanski et al., 2008; Xu et al., 2020). In our study, PM_10_ levels were most significantly increased during the wildfire period. This finding aligns with those of other studies, which also indicated that PM_10_ from wildfires markedly influences healthcare utilization. During the wildfire event, ash and larger particulate matter generated from the combustion process may have remained in the atmosphere at higher concentrations. The process of ash formation as fuel is gradually heated and combusted in a wildfire is known to be highly complex (Théry‐Parisot et al., 2010). When organic fuel undergoes complete combustion, volatile gases such as carbon dioxide (CO_2_) and carbon monoxide (CO) are produced and oxidized, leaving only mineral residues as the final byproducts. However, in most wildfire environments, oxygen supply is locally limited, leading to incomplete combustion and partial charring of some fuels. This incomplete combustion results in the formation of solid organic materials, commonly known as charcoal, as well as ash (Schmidt & Noack, 2000). Since ash consists of relatively larger particles, it is inferred that PM10 levels were observed to be higher compared to PM_2.5_. Additionally, ash can be transported from the original fire location to other areas by wind during or after the wildfire (Pereira et al., 2015). PM_10_ concentrations were likely elevated near the wildfire‐affected area, as larger particles tend to be transported by wind from the ignition point and subsequently settle in surrounding areas. Specifically, in the case of the wildfire in Samcheok, South Korea, the proximity of the monitoring station to the ignition point may have contributed to the higher observed PM_10_ concentrations. PM_10_ from wildfires contains higher levels of oxidative and proinflammatory components, which may contribute to increased toxic effects (Xu et al., 2020). Although direct comparisons are lacking, some analyses have indicated that wildfire PM_10_ increases the mortality rates from medical conditions more than PM_10_ from general ambient air pollution (urban sources) does (Black et al., 2017; Cascio, 2018; C. Liu et al., 2019; Reid, Brauer, et al., 2016). Further research is required to directly compare the public health impacts of wildfire‐generated PM_10_ with those of ambient air pollution (from urban sources) to better understand the risks.

This study had several strengths; however, it had some limitations. First, the wildfires examined were smaller in scale and shorter in duration compared to major international wildfire events, which may have led to an underestimation of their health impact (Aguilera et al., 2021; Mahsin et al., 2022). In addition, the control period was limited to the corresponding calendar weeks in 2018, which, although effective in minimizing seasonal variation and changes in population behavior, resulted in a relatively small number of control observations. This may have reduced statistical power and limited the generalizability of the findings. Nonetheless, by comparing the same period across wildfire and non‐wildfire years within the same regions, we aimed to enhance internal validity and isolate the effect of wildfire exposure. Further research involving additional control years or unaffected comparison regions would help strengthen external validity and confirm these findings in broader contexts. Second, we assessed the combined impacts of various air pollutants of wildfire smoke on healthcare utilization, and it was impossible to evaluate the effects of specific air pollutants individually. And the actual air quality monitoring stations were distanced from the wildfire ignition points, changes in air pollutants may be underestimated. This is particularly relevant for PM_2.5,_ which showed no noticeable increases, potentially causing an underestimation of the impacts of other air pollutants. Third, this study involved the analysis of Nationwide Health Insurance claims data, which was provided with certain limitations, leading to specific constraints in the analysis. The data was available only at the city level, preventing more detailed analysis at finer geographical scales. Additionally, because of the nature of claims data, it was impossible to distinguish between follow‐up visits owing to disease exacerbation and new visits owing to the onset of a new disease. By defining healthcare utilization based solely on the initial visit due to disease exacerbation, we aimed to mitigate this limitation. However, for certain respiratory diseases (e.g., asthma exacerbation), multiple visits are expected, which may lead to an underestimation of the overall healthcare burden with this approach. Finally, while this study minimized seasonal and environmental confounding by comparing healthcare utilization during the same period in the same region across 2017 and 2018, certain unmeasured confounders—such as changes in population behavior or air quality variations unrelated to wildfires—could not be fully accounted for due to data limitations. Moreover, temperature, a well‐known modifier of respiratory outcomes, was not included in the final model. This exclusion was based on its minimal variation during the study periods, its complex interaction with wildfire smoke, and strong collinearity with wildfire‐related pollutants, which reduced model stability. Although we acknowledge this as a limitation, including temperature would have compromised the interpretability of wildfire‐specific effects, and a meaningful sensitivity analysis was not feasible with the available data. These limitations highlight the need for future studies using more granular environmental data and advanced modeling techniques to better isolate the health effects of wildfire smoke under varying meteorological conditions.

## 5 Conclusion

Wildfires in South Korea (even short‐term exposure to small wildfires) significantly increase the risk of healthcare utilization for respiratory diseases. Exposure to wildfire smoke had immediate and delayed effects on the respiratory diseases associated with healthcare utilization in children aged <9 years and older adults. These findings underscore the acute and lingering health risks posed by wildfires, emphasizing the need for enhanced public health measures and medical preparedness in wildfire‐prone regions to mitigate adverse health effects on vulnerable populations. Satellite technology aids in effectively identifying and monitoring areas that are vulnerable to wildfire smoke, facilitating targeted interventions to mitigate respiratory health risks during future wildfire events.

## Conflict of Interest

The authors declare no conflicts of interest relevant to this study.

## Supporting information

Supporting Information S1

## Data Availability

Data supporting this research are available from the National Health Insurance Service of South Korea (https://nhiss.nhis.or.kr/), and are not accessible to the public or research community. To gain access to the database, researchers must submit data‐request application with a study protocol with approval from an Institutional Review Board.

## References

[gh270100-bib-0001] Adetona, O. , Reinhardt, T. E. , Domitrovich, J. , Broyles, G. , Adetona, A. M. , Kleinman, M. T. , et al. (2016). Review of the health effects of wildland fire smoke on wildland firefighters and the public. Inhalation Toxicology, 28(3), 95–139. 10.3109/08958378.2016.1145771 26915822

[gh270100-bib-0002] Aguilera, R. , Corringham, T. , Gershunov, A. , & Benmarhnia, T. (2021). Wildfire smoke impacts respiratory health more than fine particles from other sources: Observational evidence from Southern California. Nature Communications, 12(1), 1493. 10.1038/s41467-021-21708-0 PMC7935892 33674571

[gh270100-bib-0003] Alman, B. L. , Pfister, G. , Hao, H. , Stowell, J. , Hu, X. , Liu, Y. , & Strickland, M. J. (2016). The association of wildfire smoke with respiratory and cardiovascular emergency department visits in Colorado in 2012: A case crossover study. Environmental Health, 15(1), 64. 10.1186/s12940-016-0146-8 27259511 PMC4893210

[gh270100-bib-0004] Altman, D. G. , & Bland, J. M. (2003). Interaction revisited: The difference between two estimates. BMJ, 326(7382), 219. 10.1136/bmj.326.7382.219 12543843 PMC1125071

[gh270100-bib-0005] Bhaskaran, K. , Gasparrini, A. , Hajat, S. , Smeeth, L. , & Armstrong, B. (2013). Time series regression studies in environmental epidemiology. International Journal of Epidemiology, 42(4), 1187–1195. 10.1093/ije/dyt092 23760528 PMC3780998

[gh270100-bib-0006] Black, C. , Tesfaigzi, Y. , Bassein, J. A. , & Miller, L. A. (2017). Wildfire smoke exposure and human health: Significant gaps in research for a growing public health issue. Environmental Toxicology and Pharmacology, 55, 186–195. 10.1016/j.etap.2017.08.022 28892756 PMC5628149

[gh270100-bib-0007] Blando, J. , Allen, M. , Galadima, H. , Tolson, T. , Akpinar‐Elci, M. , & Szklo‐Coxe, M. (2022). Observations of delayed changes in respiratory function among allergy clinic patients exposed to wildfire smoke. International Journal of Environmental Research and Public Health, 19(3), 1241. 10.3390/ijerph19031241 35162264 PMC8835059

[gh270100-bib-0008] Borchers Arriagada, N. , Horsley, J. A. , Palmer, A. J. , Morgan, G. G. , Tham, R. , & Johnston, F. H. (2019). Association between fire smoke fine particulate matter and asthma‐related outcomes: Systematic review and meta‐analysis. Environmental Research, 179(Pt A), 108777. 10.1016/j.envres.2019.108777 31593836

[gh270100-bib-0009] Bowman, W. S. , Schmidt, R. J. , Sanghar, G. K. , Thompson Iii, G. R. , Ji, H. , Zeki, A. A. , & Haczku, A. (2024). “Air That Once Was Breath” Part 1: Wildfire‐smoke‐induced mechanisms of airway inflammation—“Climate change, allergy and immunology” Special IAAI article collection: Collegium internationale allergologicum update 2023. International Archives of Allergy and Immunology, 185(6), 600–616. 10.1159/000536578 38452750 PMC11487202

[gh270100-bib-0010] Cascio, W. E. (2018). Wildland fire smoke and human health. Science of the Total Environment, 624, 586–595. 10.1016/j.scitotenv.2017.12.086 29272827 PMC6697173

[gh270100-bib-0011] Delfino, R. J. , Brummel, S. , Wu, J. , Stern, H. , Ostro, B. , Lipsett, M. , et al. (2009). The relationship of respiratory and cardiovascular hospital admissions to the southern California wildfires of 2003. Occupational and Environmental Medicine, 66(3), 189–197. 10.1136/oem.2008.041376 19017694 PMC4176821

[gh270100-bib-0012] Dong, T. T. , Hinwood, A. L. , Callan, A. C. , Zosky, G. , & Stock, W. D. (2017). In vitro assessment of the toxicity of bushfire emissions: A review. Science of the Total Environment, 603, 268–278. 10.1016/j.scitotenv.2017.06.062 28628818

[gh270100-bib-0013] dos Santos, L. R. , Alves‐Correia, M. , Câmara, M. , Lélis, M. , Caldeira, C. , da Luz Brazão, M. , & Nóbrega, J. J. (2018). Multiple victims of carbon monoxide poisoning in the aftermath of a wildfire: A case series. Acta Medica Portuguesa, 31(3), 146–151. 10.20344/amp.9811 29790465

[gh270100-bib-0014] Gan, R. W. , Ford, B. , Lassman, W. , Pfister, G. , Vaidyanathan, A. , Fischer, E. , et al. (2017). Comparison of wildfire smoke estimation methods and associations with cardiopulmonary‐related hospital admissions. GeoHealth, 1(3), 122–136. 10.1002/2017gh000073 28868515 PMC5580836

[gh270100-bib-0015] Haikerwal, A. , Akram, M. , Sim, M. R. , Meyer, M. , Abramson, M. J. , & Dennekamp, M. (2016). Fine particulate matter (PM_2.5_) exposure during a prolonged wildfire period and emergency department visits for asthma. Respirology, 21(1), 88–94. 10.1111/resp.12613 26346113

[gh270100-bib-0016] Horne, B. D. , Joy, E. A. , Hofmann, M. G. , Gesteland, P. H. , Cannon, J. B. , Lefler, J. S. , et al. (2018). Short‐term elevation of fine particulate matter air pollution and acute lower respiratory infection. American Journal of Respiratory and Critical Care Medicine, 198(6), 759–766. 10.1164/rccm.201709-1883oc 29652174

[gh270100-bib-0017] Hutchinson, J. A. , Vargo, J. , Milet, M. , French, N. H. , Billmire, M. , Johnson, J. , & Hoshiko, S. (2018). The San Diego 2007 wildfires and Medi‐Cal emergency department presentations, inpatient hospitalizations, and outpatient visits: An observational study of smoke exposure periods and a bidirectional case‐crossover analysis. PLoS Medicine, 15(7), e1002601. 10.1371/journal.pmed.1002601 29990362 PMC6038982

[gh270100-bib-0018] Jang, E. , Kang, Y. , Im, J. , Lee, D.‐W. , Yoon, J. , & Kim, S.‐K. (2019). Detection and monitoring of forest fires using Himawari‐8 geostationary satellite data in South Korea. Remote Sensing, 11(3), 271. 10.3390/rs11030271

[gh270100-bib-0019] Jia, C. , Sun, L. , Zhang, X. , & Wang, Y. (2020). Verification of MCD19A2 data and study of aerosol characteristics in Beijing‐Tianjin‐Hebei region. In ISPRS Annals of the Photogrammetry, Remote Sensing and Spatial Information Sciences (Vol. V‐3‐2020, pp. 675–679). 10.5194/isprs-annals-V-3-2020-675-2020

[gh270100-bib-0020] Kim, D. S. , & Lee, Y. W. (2016). Retrieval of fire radiative power from Himawari‐8 satellite data using the mid‐infrared radiance method [Retrieval of Fire Radiative Power from Himawari‐8 Satellite Data Using the Mid‐Infrared Radiance Method]. Journal of Korean Society for Geospatial Information Science, 24(4), 105–113. 10.7319/kogsis.2016.24.4.105

[gh270100-bib-0021] Kim, Y. H. , Warren, S. H. , Krantz, Q. T. , King, C. , Jaskot, R. , Preston, W. T. , et al. (2018). Mutagenicity and lung toxicity of smoldering vs. flaming emissions from various biomass fuels: Implications for health effects from wildland fires. Environmental Health Perspectives, 126(1), 017011. 10.1289/ehp2200 29373863 PMC6039157

[gh270100-bib-0022] Kondo, M. C. , De Roos, A. J. , White, L. S. , Heilman, W. E. , Mockrin, M. H. , Gross‐Davis, C. A. , & Burstyn, I. (2019). Meta‐analysis of heterogeneity in the effects of wildfire smoke exposure on respiratory health in North America. International Journal of Environmental Research and Public Health, 16(6), 960. 10.3390/ijerph16060960 30889810 PMC6466235

[gh270100-bib-0065] Korea Forest Service . (2020). Forest fire statistical yearbook 2019. Korea Forest Service. (In Korean).

[gh270100-bib-0023] Korea Meteorological Administration . (2024). Retrieved from http://data.kma.go.kr

[gh270100-bib-0024] Korean Statistical Information Service . (2024). KOSIS. Retrieved from https://kosis.kr

[gh270100-bib-0025] Landguth, E. L. , Holden, Z. A. , Graham, J. , Stark, B. , Mokhtari, E. B. , Kaleczyc, E. , et al. (2020). The delayed effect of wildfire season particulate matter on subsequent influenza season in a mountain west region of the USA. Environment International, 139, 105668. 10.1016/j.envint.2020.105668 32244099 PMC7275907

[gh270100-bib-0026] Lee, S.‐J. , Jiwon, K. I. M. , Kim, K.‐J. , & Yeongho, K. I. M. (2017). Development of FBI(Fire Burn Index) for Sentinel‐2 images and an experiment for detection of burned areas in Korea. Journal of the Association of Korean Photo‐Geographers, 27, 187–202. 10.35149/jakpg.2017.27.4.012

[gh270100-bib-0027] Li, B. , Yuan, H. , Feng, N. , & Tao, S. (2010). Spatial and temporal variations of aerosol optical depth in China during the period from 2003 to 2006. International Journal of Remote Sensing, 31(7), 1801–1817. 10.1080/01431160902926665

[gh270100-bib-0028] Liu, C. , Chen, R. , Sera, F. , Vicedo‐Cabrera, A. M. , Guo, Y. , Tong, S. , et al. (2019). Ambient particulate air pollution and daily mortality in 652 cities. New England Journal of Medicine, 381(8), 705–715. 10.1056/NEJMoa1817364 31433918 PMC7891185

[gh270100-bib-0029] Liu, J. C. , Pereira, G. , Uhl, S. A. , Bravo, M. A. , & Bell, M. L. (2015). A systematic review of the physical health impacts from non‐occupational exposure to wildfire smoke. Environmental Research, 136, 120–132. 10.1016/j.envres.2014.10.015 25460628 PMC4262561

[gh270100-bib-0030] Liu, J. C. , Wilson, A. , Mickley, L. J. , Dominici, F. , Ebisu, K. , Wang, Y. , et al. (2017). Wildfire‐specific fine particulate matter and risk of hospital admissions in urban and rural counties. Epidemiology, 28(1), 77–85. 10.1097/ede.0000000000000556 27648592 PMC5130603

[gh270100-bib-0031] Lowery, E. M. , Brubaker, A. L. , Kuhlmann, E. , & Kovacs, E. J. (2013). The aging lung. Clinical Interventions in Aging, 8, 1489–1496. 10.2147/cia.S51152 24235821 PMC3825547

[gh270100-bib-0032] Mahsin, M. , Cabaj, J. , & Saini, V. (2022). Respiratory and cardiovascular condition‐related physician visits associated with wildfire smoke exposure in Calgary, Canada, in 2015: A population‐based study. International Journal of Epidemiology, 51(1), 166–178. 10.1093/ije/dyab206 34561694

[gh270100-bib-0033] Makkonen, U. , Hellén, H. , Anttila, P. , & Ferm, M. (2010). Size distribution and chemical composition of airborne particles in south‐eastern Finland during different seasons and wildfire episodes in 2006. Science of the Total Environment, 408(3), 644–651. 10.1016/j.scitotenv.2009.10.050 19903567

[gh270100-bib-0034] Masson‐Delmotte, V. , Zhai, P. , Pörtner, H. , Roberts, D. , Skea, J. , Shukla, P. , et al. (2018). Global warming of 1.5 C: Special report. Intergovernmental Panel on Climate Change.

[gh270100-bib-0035] Nakayama Wong, L. S. , Aung, H. H. , Lamé, M. W. , Wegesser, T. C. , & Wilson, D. W. (2011). Fine particulate matter from urban ambient and wildfire sources from California's San Joaquin Valley initiate differential inflammatory, oxidative stress, and xenobiotic responses in human bronchial epithelial cells. Toxicology in Vitro, 25(8), 1895–1905. 10.1016/j.tiv.2011.06.001 21703343

[gh270100-bib-0036] Pereira, P. , Cerdà, A. , Úbeda, X. , Mataix‐Solera, J. , Arcenegui, V. , & Zavala, L. M. (2015). Modelling the impacts of wildfire on ash thickness in a short‐term period. Land Degradation & Development, 26(2), 180–192. 10.1002/ldr.2195

[gh270100-bib-0037] Reid, C. E. , Brauer, M. , Johnston, F. H. , Jerrett, M. , Balmes, J. R. , & Elliott, C. T. (2016). Critical review of health impacts of wildfire smoke exposure. Environmental Health Perspectives, 124(9), 1334–1343. 10.1289/ehp.1409277 27082891 PMC5010409

[gh270100-bib-0038] Reid, C. E. , Considine, E. M. , Watson, G. L. , Telesca, D. , Pfister, G. G. , & Jerrett, M. (2019). Associations between respiratory health and ozone and fine particulate matter during a wildfire event. Environment International, 129, 291–298. 10.1016/j.envint.2019.04.033 31146163

[gh270100-bib-0039] Reid, C. E. , Jerrett, M. , Tager, I. B. , Petersen, M. L. , Mann, J. K. , & Balmes, J. R. (2016). Differential respiratory health effects from the 2008 northern California wildfires: A spatiotemporal approach. Environmental Research, 150, 227–235. 10.1016/j.envres.2016.06.012 27318255

[gh270100-bib-0040] Reid, C. E. , & Maestas, M. M. (2019). Wildfire smoke exposure under climate change: Impact on respiratory health of affected communities. Current Opinion in Pulmonary Medicine, 25(2), 179–187. 10.1097/mcp.0000000000000552 30461534 PMC6743728

[gh270100-bib-0041] Roscioli, E. , Hamon, R. , Lester, S. E. , Jersmann, H. P. A. , Reynolds, P. N. , & Hodge, S. (2018). Airway epithelial cells exposed to wildfire smoke extract exhibit dysregulated autophagy and barrier dysfunction consistent with COPD. Respiratory Research, 19(1), 234. 10.1186/s12931-018-0945-2 30486816 PMC6263553

[gh270100-bib-0042] Saim, A. A. , & Aly, M. H. (2024). Big data analyses for determining the spatio‐temporal trends of air pollution due to wildfires in California using Google Earth Engine. Atmospheric Pollution Research, 15(9), 102226. 10.1016/j.apr.2024.102226

[gh270100-bib-0043] Schmidt, M. W. I. , & Noack, A. G. (2000). Black carbon in soils and sediments: Analysis, distribution, implications, and current challenges. Global Biogeochemical Cycles, 14(3), 777–793. 10.1029/1999GB001208

[gh270100-bib-0044] Schneider, J. L. , Rowe, J. H. , Garcia‐de‐Alba, C. , Kim, C. F. , Sharpe, A. H. , & Haigis, M. C. (2021). The aging lung: Physiology, disease, and immunity. Cell, 184(8), 1990–2019. 10.1016/j.cell.2021.03.005 33811810 PMC8052295

[gh270100-bib-0045] Schwarz, L. , Aguilera, R. , Aguilar‐Dodier, L. C. , Castillo Quiñones, J. E. , García, M. E. A. , & Benmarhnia, T. (2023). Wildfire smoke knows no borders: Differential vulnerability to smoke effects on cardio‐respiratory health in the San Diego‐Tijuana region. PLOS Global Public Health, 3(6), e0001886. 10.1371/journal.pgph.0001886 37347761 PMC10287006

[gh270100-bib-0047] Sim, S. , Kim, W. , Lee, J. , Kang, Y. , Im, J. , Kwon, C. , & Kim, S. (2020). Wildfire severity mapping using sentinel satellite data based on machine learning approaches. Korean Journal of Remote Sensing, 36(5_3), 1109–1123.

[gh270100-bib-0048] Stone, S. L. , Anderko, L. , Berger, M. , Butler, C. R. , Cascio, W. E. , Clune, A. , et al. (2019). Wildfire smoke: A guide for public health officials. US Environmental Protection Agency.

[gh270100-bib-0049] Su, C. , Hampel, R. , Franck, U. , Wiedensohler, A. , Cyrys, J. , Pan, X. , et al. (2015). Assessing responses of cardiovascular mortality to particulate matter air pollution for pre‐during‐ and post‐2008 Olympics periods. Environmental Research, 142, 112–122. 10.1016/j.envres.2015.06.025 26133808

[gh270100-bib-0050] Sun, Q. , Miao, C. , Hanel, M. , Borthwick, A. G. , Duan, Q. , Ji, D. , & Li, H. (2019). Global heat stress on health, wildfires, and agricultural crops under different levels of climate warming. Environment International, 128, 125–136. 10.1016/j.envint.2019.04.025 31048130

[gh270100-bib-0051] Tao, Z. , He, H. , Sun, C. , Tong, D. , & Liang, X.‐Z. (2020). Impact of fire emissions on US air quality from 1997 to 2016–a modeling study in the satellite era. Remote Sensing, 12(6), 913. 10.3390/rs12060913

[gh270100-bib-0052] Théry‐Parisot, I. , Chabal, L. , & Chrzavzez, J. (2010). Anthracology and taphonomy, from wood gathering to charcoal analysis. A review of the taphonomic processes modifying charcoal assemblages, in archaeological contexts. Palaeogeography, Palaeoclimatology, Palaeoecology, 291(1), 142–153. 10.1016/j.palaeo.2009.09.016

[gh270100-bib-0053] Urbanski, S. P. , Hao, W. M. , & Baker, S. (2008). Chemical composition of wildland fire emissions. In Developments in Environmental Science (Vol. 8, pp. 79–107).

[gh270100-bib-0054] Verma, V. , Polidori, A. , Schauer, J. J. , Shafer, M. M. , Cassee, F. R. , & Sioutas, C. (2009). Physicochemical and toxicological profiles of particulate matter in Los Angeles during the October 2007 southern California wildfires. Environmental Science & Technology, 43(3), 954–960. 10.1021/es8021667 19245042

[gh270100-bib-0055] Wegesser, T. C. , Pinkerton, K. E. , & Last, J. A. (2009). California wildfires of 2008: Coarse and fine particulate matter toxicity. Environmental Health Perspectives, 117(6), 893–897. 10.1289/ehp.0800166 19590679 PMC2702402

[gh270100-bib-0056] Wei, X. , Chang, N.‐B. , Bai, K. , & Gao, W. (2020). Satellite remote sensing of aerosol optical depth: Advances, challenges, and perspectives. Critical Reviews in Environmental Science and Technology, 50(16), 1640–1725. 10.1080/10643389.2019.1665944

[gh270100-bib-0057] Wettstein, Z. S. , Hoshiko, S. , Fahimi, J. , Harrison, R. J. , Cascio, W. E. , & Rappold, A. G. (2018). Cardiovascular and cerebrovascular emergency department visits associated with wildfire smoke exposure in California in 2015. Journal of American Heart Association, 7(8), e007492. 10.1161/jaha.117.007492 PMC6015400 29643111

[gh270100-bib-0058] Williams, K. M. , Franzi, L. M. , & Last, J. A. (2013). Cell‐specific oxidative stress and cytotoxicity after wildfire coarse particulate matter instillation into mouse lung. Toxicology and Applied Pharmacology, 266(1), 48–55. 10.1016/j.taap.2012.10.017 23142465 PMC3546532

[gh270100-bib-0059] Wotton, B. M. , Flannigan, M. D. , & Marshall, G. A. (2017). Potential climate change impacts on fire intensity and key wildfire suppression thresholds in Canada. Environmental Research Letters, 12(9), 095003. 10.1088/1748-9326/aa7e6e

[gh270100-bib-0060] Xia, X. , Chen, H. , Goloub, P. , Zhang, W. , Chatenet, B. , & Wang, P. (2007). A compilation of aerosol optical properties and calculation of direct radiative forcing over an urban region in northern China. Journal of Geophysical Research, 112(D12). 10.1029/2006JD008119

[gh270100-bib-0061] Xu, R. , Yu, P. , Abramson Michael, J. , Johnston Fay, H. , Samet Jonathan, M. , Bell Michelle, L. , et al. (2020). Wildfires, global climate change, and human health. New England Journal of Medicine, 383(22), 2173–2181. 10.1056/NEJMsr2028985 33034960

[gh270100-bib-0062] Youssouf, H. , Liousse, C. , Roblou, L. , Assamoi, E.‐M. , Salonen, R. O. , Maesano, C. , et al. (2014). Non‐accidental health impacts of wildfire smoke. International Journal of Environmental Research and Public Health, 11(11), 11772–11804. 10.3390/ijerph111111772 25405597 PMC4245643

[gh270100-bib-0063] Zhang, Y. , Tingting, Y. , Huang, W. , Yu, P. , Chen, G. , Xu, R. , et al. (2024). Health impacts of wildfire smoke on children and adolescents: A systematic review and meta‐analysis. Current Environmental Health Reports, 11(1), 46–60. 10.1007/s40572-023-00420-9 38038861

[gh270100-bib-0064] Zhu, Z. , Zhang, Z. , Liu, F. , Chen, Z. , Ren, Y. , & Guo, Q. (2023). Study on accuracy evaluation of MCD19A2 and spatiotemporal distribution of AOD in arid zones of Central Asia. Sustainability, 15(18), 13959. 10.3390/su151813959

