# Supplementary material for: Impact of Wildfire Smoke on Respiratory Disease Associated Healthcare Utilization in Gang‐Won Province, South Korea, in 2017
Source: Geohealth. 2026 Jan 28;10(2):e2025GH001332. doi: 10.1029/2025GH001332 (PMC12852965; doi:10.1029/2025GH001332)
Supplement: Supplementary file 1 — Supporting Information S1 [file GH2-10-e2025GH001332-s001.docx]

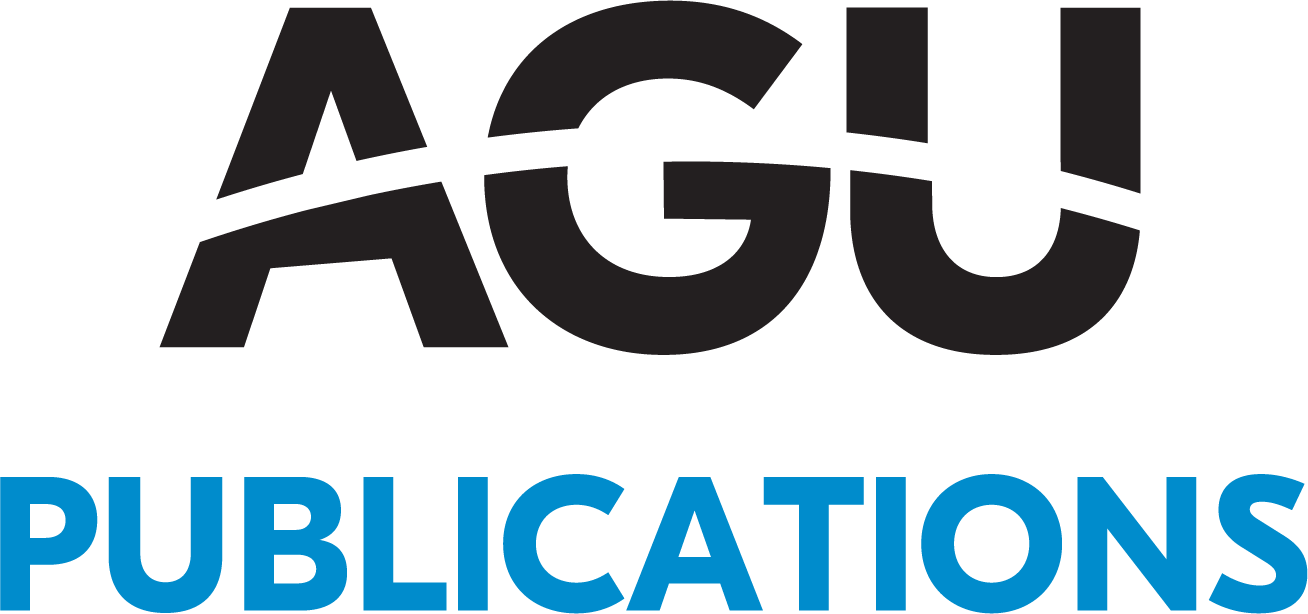


*GeoHealth*

Supporting Information for

**Impact of wildfire smoke on respiratory disease associated healthcare utilization in Gang-won province, South Korea, in 2017**

Min-Taek Lee^1,2^, Hoyoung Cha^6^, Ju Won Lee^1,2^, Jongjin Baik^7^, Hae In Jung^3,4^, Kyoung Min Moon^3,4^, Changhyun Jun^7^, Sun-Young Jung^1,2^ Kang-Mo Gu^3,4,5^

^1^College of Pharmacy, Chung-Ang University, Seoul, Korea, ^2^Department of Global Innovative Drugs, The Graduate School of Chung-Ang University, Seoul, Korea, ^3^Department of Internal Medicine, College of Medicine, Chung-Ang University, Seoul, South Korea, ^4^Division of Pulmonary and Allergy Medicine, Department of Internal Medicine, Chung-Ang University Hospital, Seoul, Korea, ^5^Biomedical Research Institute, Chung-Ang University Hospital, Seoul, South Korea, ^6^Department of Civil, Environmental and Architectural Engineering, Korea University, Seoul, Korea, ^7^School of Civil, Environmental and Architectural Engineering, Korea University, Seoul, Korea

**Contents of this file**

Text S1 to S3

**Table S1.** Model Fitting Test Results According to Lag Days of Wildfire Exposure (AIC, AICC, BIC)

|  |  | **Samcheok** | **Donghae** | **Samcheok, Donghae** |
| --- | --- | --- | --- | --- |
| Lag 0 | AIC | 6245.6354 | 7912.895 | 13364.325 |
|  | AICC | 6254.9688 | 7922.2283 | 13373.658 |
|  | BIC | 6274.9562 | 7942.2158 | 13393.646 |
| Lag 1 | AIC | 6150.6494 | 8690.888 | 14125.778 |
|  | AICC | 6159.9827 | 8700.2213 | 14135.111 |
|  | BIC | 6179.9702 | 8720.2088 | 14155.099 |
| Lag 2 | AIC | 5358.416 | 6902.6713 | 11769.988 |
|  | AICC | 5367.7493 | 6912.0046 | 11779.322 |
|  | BIC | 5387.7368 | 6931.9921 | 11799.309 |
| Lag 3 | AIC | 5991.2877 | 7140.1932 | 12555.448 |
|  | AICC | 6000.621 | 7149.5266 | 12564.781 |
|  | BIC | 6020.6085 | 7169.5141 | 12584.768 |
| A three-day  moving average | AIC | 5430.9938 | 6651.2338 | 11351.582 |
|  | AICC | 5440.3272 | 6660.5672 | 11360.915 |
|  | BIC | 5460.3146 | 6680.5546 | 11380.902 |

AIC (Akaike Information Criterion), AICC (Corrected Akaike Information Criterion), BIC (Bayes Information Criterion)

**Table S2.** Frequency and Incidence of Respiratory Disease Healthcare Utilization by Type (hospitalization, outpatient department, emergency department visit) in Wildfire-Exposed Areas

**(a) Samcheok (Direct-exposure area)**

| Age | Pre-wildfire Period | | | | Wildfire Period | | | | Immediate post-wildfire period | | | | Extended post-wildfire period | | | |
| --- | --- | --- | --- | --- | --- | --- | --- | --- | --- | --- | --- | --- | --- | --- | --- | --- |
| Groups | Count | Pop* | PY | IR | Count | Pop* | PY | IR | Count | Pop* | PY | IR | Count | Pop* | PY | IR |
| H |  |  |  |  |  |  |  |  |  |  |  |  |  |  |  |  |
| All ages | 339 | 69,212 | 968,968 | 0.0003 | 73 | 69,212 | 276,848 | 0.0003 | 270 | 69,212 | 968,968 | 0.0003 | 352 | 69,212 | 968,968 | 0.0004 |
| 0–9 | 18 | 4,459 | 62,426 | 0.0003 | 8 | 4,459 | 17,836 | 0.0004 | 20 | 4,459 | 62,426 | 0.0003 | 13 | 4,459 | 62,426 | 0.0002 |
| 10–19 | 15 | 6,910 | 96,740 | 0.0002 |  | 6,910 | 27,640 | 0.0000 | 6 | 6,910 | 96,740 | 0.0001 | 10 | 6,910 | 96,740 | 0.0001 |
| 20–29 | 7 | 8,477 | 118,678 | 0.0001 | 2 | 8,477 | 33,908 | 0.0001 | 12 | 8,477 | 118,678 | 0.0001 | 10 | 8,477 | 118,678 | 0.0001 |
| 30–39 | 16 | 6,888 | 96,432 | 0.0002 | 3 | 6,888 | 27,552 | 0.0001 | 18 | 6,888 | 96,432 | 0.0002 | 12 | 6,888 | 96,432 | 0.0001 |
| 40–49 | 20 | 10,102 | 141,428 | 0.0001 | 7 | 10,102 | 40,408 | 0.0002 | 21 | 10,102 | 141,428 | 0.0001 | 24 | 10,102 | 141,428 | 0.0002 |
| 50–59 | 40 | 12,062 | 168,868 | 0.0002 | 10 | 12,062 | 48,248 | 0.0002 | 40 | 12,062 | 168,868 | 0.0002 | 54 | 12,062 | 168,868 | 0.0003 |
| 60–69 | 55 | 9,527 | 133,378 | 0.0004 | 13 | 9,527 | 38,108 | 0.0003 | 59 | 9,527 | 133,378 | 0.0004 | 57 | 9,527 | 133,378 | 0.0004 |
| 70–79 | 81 | 7,013 | 98,182 | 0.0008 | 14 | 7,013 | 28,052 | 0.0005 | 54 | 7,013 | 98,182 | 0.0005 | 88 | 7,013 | 98,182 | 0.0009 |
| ≥80 | 87 | 3,297 | 46,158 | 0.0019 | 16 | 3,297 | 13,188 | 0.0012 | 40 | 3,297 | 46,158 | 0.0009 | 84 | 3,297 | 46,158 | 0.0018 |
| O |  |  |  |  |  |  |  |  |  |  |  |  |  |  |  |  |
| All ages | 8,161 | 69,212 | 968,968 | 0.0084 | 2,978 | 69,212 | 276,848 | 0.0108 | 8,500 | 69,212 | 968,968 | 0.0088 | 8,011 | 69,212 | 968,968 | 0.0083 |
| 0–9 | 894 | 4,459 | 62,426 | 0.0143 | 343 | 4,459 | 17,836 | 0.0192 | 832 | 4,459 | 62,426 | 0.0133 | 869 | 4,459 | 62,426 | 0.0139 |
| 10–19 | 489 | 6,910 | 96,740 | 0.0051 | 151 | 6,910 | 27,640 | 0.0055 | 426 | 6,910 | 96,740 | 0.0044 | 404 | 6,910 | 96,740 | 0.0042 |
| 20–29 | 497 | 8,477 | 118,678 | 0.0042 | 178 | 8,477 | 33,908 | 0.0052 | 487 | 8,477 | 118,678 | 0.0041 | 410 | 8,477 | 118,678 | 0.0035 |
| 30–39 | 541 | 6,888 | 96,432 | 0.0056 | 198 | 6,888 | 27,552 | 0.0072 | 590 | 6,888 | 96,432 | 0.0061 | 516 | 6,888 | 96,432 | 0.0054 |
| 40–49 | 716 | 10,102 | 141,428 | 0.0051 | 244 | 10,102 | 40,408 | 0.0060 | 779 | 10,102 | 141,428 | 0.0055 | 720 | 10,102 | 141,428 | 0.0051 |
| 50–59 | 1,276 | 12,062 | 168,868 | 0.0076 | 451 | 12,062 | 48,248 | 0.0093 | 1,362 | 12,062 | 168,868 | 0.0081 | 1,254 | 12,062 | 168,868 | 0.0074 |
| 60–69 | 1,512 | 9,527 | 133,378 | 0.0113 | 567 | 9,527 | 38,108 | 0.0149 | 1,660 | 9,527 | 133,378 | 0.0124 | 1,556 | 9,527 | 133,378 | 0.0117 |
| 70–79 | 1,476 | 7,013 | 98,182 | 0.0150 | 592 | 7,013 | 28,052 | 0.0211 | 1,563 | 7,013 | 98,182 | 0.0159 | 1,524 | 7,013 | 98,182 | 0.0155 |
| ≥80 | 760 | 3,297 | 46,158 | 0.0165 | 254 | 3,297 | 13,188 | 0.0193 | 801 | 3,297 | 46,158 | 0.0174 | 758 | 3,297 | 46,158 | 0.0164 |
| E |  |  |  |  |  |  |  |  |  |  |  |  |  |  |  |  |
| All ages | 559 | 69,212 | 968,968 | 0.0006 | 164 | 69,212 | 276,848 | 0.0006 | 453 | 69,212 | 968,968 | 0.0005 | 519 | 69,212 | 968,968 | 0.0005 |
| 0–9 | 52 | 4,459 | 62,426 | 0.0008 | 15 | 4,459 | 17,836 | 0.0008 | 41 | 4,459 | 62,426 | 0.0007 | 38 | 4,459 | 62,426 | 0.0006 |
| 10–19 | 34 | 6,910 | 96,740 | 0.0004 | 8 | 6,910 | 27,640 | 0.0003 | 25 | 6,910 | 96,740 | 0.0003 | 27 | 6,910 | 96,740 | 0.0003 |
| 20–29 | 32 | 8,477 | 118,678 | 0.0003 | 17 | 8,477 | 33,908 | 0.0005 | 31 | 8,477 | 118,678 | 0.0003 | 28 | 8,477 | 118,678 | 0.0002 |
| 30–39 | 40 | 6,888 | 96,432 | 0.0004 | 12 | 6,888 | 27,552 | 0.0004 | 34 | 6,888 | 96,432 | 0.0004 | 39 | 6,888 | 96,432 | 0.0004 |
| 40–49 | 57 | 10,102 | 141,428 | 0.0004 | 17 | 10,102 | 40,408 | 0.0004 | 43 | 10,102 | 141,428 | 0.0003 | 43 | 10,102 | 141,428 | 0.0003 |
| 50–59 | 73 | 12,062 | 168,868 | 0.0004 | 26 | 12,062 | 48,248 | 0.0005 | 88 | 12,062 | 168,868 | 0.0005 | 88 | 12,062 | 168,868 | 0.0005 |
| 60–69 | 82 | 9,527 | 133,378 | 0.0006 | 30 | 9,527 | 38,108 | 0.0008 | 83 | 9,527 | 133,378 | 0.0006 | 78 | 9,527 | 133,378 | 0.0006 |
| 70–79 | 95 | 7,013 | 98,182 | 0.0010 | 21 | 7,013 | 28,052 | 0.0007 | 62 | 7,013 | 98,182 | 0.0006 | 93 | 7,013 | 98,182 | 0.0009 |
| ≥80 | 94 | 3,297 | 46,158 | 0.0020 | 18 | 3,297 | 13,188 | 0.0014 | 46 | 3,297 | 46,158 | 0.0010 | 85 | 3,297 | 46,158 | 0.0018 |

H = hospitalization; O = outpatient clinic visit; E = emergency depart visit

Pop = total population of wildfire-exposed areas; PY = person-years; IR = incidence rate

Pre-wildfire (April 22, 2017, to May 5, 2017), wildfire (May 6, 2017, to May 9, 2017), immediate post-wildfire (May 10, 2017, to May 23, 2017), and extended post-wildfire (May 24, 2017, to June 6, 2017) periods

**(b) Donghae (Indirect-exposure area)**

| Age | Pre-wildfire Period | | | | Wildfire Period | | | | Immediate post-wildfire period | | | | Extended post-wildfire period | | | |
| --- | --- | --- | --- | --- | --- | --- | --- | --- | --- | --- | --- | --- | --- | --- | --- | --- |
| Groups | Count | Pop* | PY | IR | Count | Pop* | PY | IR | Count | Pop* | PY | IR | Count | Pop* | PY | IR |
| H |  |  |  |  |  |  |  |  |  |  |  |  |  |  |  |  |
| All ages | 399 | 92,814 | 1,299,396 | 0.0003 | 84 | 92,814 | 371,256 | 0.0002 | 339 | 92,814 | 1,299,396 | 0.0003 | 450 | 92,814 | 1,299,396 | 0.0003 |
| 0–9 | 22 | 7,838 | 109,732 | 0.0002 | 10 | 7,838 | 31,352 | 0.0003 | 34 | 7,838 | 109,732 | 0.0003 | 42 | 7,838 | 109,732 | 0.0004 |
| 10–19 | 6 | 10,177 | 142,478 | 0.0000 | 2 | 10,177 | 40,708 | 0.0000 | 15 | 10,177 | 142,478 | 0.0001 | 9 | 10,177 | 142,478 | 0.0001 |
| 20–29 | 7 | 9,572 | 134,008 | 0.0001 | 3 | 9,572 | 38,288 | 0.0001 | 6 | 9,572 | 134,008 | 0.0000 | 17 | 9,572 | 134,008 | 0.0001 |
| 30–39 | 21 | 11,302 | 158,228 | 0.0001 | 1 | 11,302 | 45,208 | 0.0000 | 22 | 11,302 | 158,228 | 0.0001 | 27 | 11,302 | 158,228 | 0.0002 |
| 40–49 | 31 | 15,843 | 221,802 | 0.0001 | 9 | 15,843 | 63,372 | 0.0001 | 36 | 15,843 | 221,802 | 0.0002 | 30 | 15,843 | 221,802 | 0.0001 |
| 50–59 | 71 | 15,835 | 221,690 | 0.0003 | 19 | 15,835 | 63,340 | 0.0003 | 69 | 15,835 | 221,690 | 0.0003 | 71 | 15,835 | 221,690 | 0.0003 |
| 60–69 | 80 | 11,446 | 160,244 | 0.0005 | 11 | 11,446 | 45,784 | 0.0002 | 61 | 11,446 | 160,244 | 0.0004 | 89 | 11,446 | 160,244 | 0.0006 |
| 70–79 | 93 | 7,466 | 104,524 | 0.0009 | 20 | 7,466 | 29,864 | 0.0007 | 60 | 7,466 | 104,524 | 0.0006 | 85 | 7,466 | 104,524 | 0.0008 |
| ≥80 | 68 | 2,929 | 41,006 | 0.0017 | 9 | 2,929 | 11,716 | 0.0008 | 36 | 2,929 | 41,006 | 0.0009 | 80 | 2,929 | 41,006 | 0.0020 |
| O |  |  |  |  |  |  |  |  |  |  |  |  |  |  |  |  |
| All ages | 11,504 | 92,814 | 1,299,396 | 0.0089 | 4,302 | 92,814 | 371,256 | 0.0116 | 11,937 | 92,814 | 1,299,396 | 0.0092 | 11,071 | 92,814 | 1,299,396 | 0.0085 |
| 0–9 | 1,602 | 7,838 | 109,732 | 0.0146 | 596 | 7,838 | 31,352 | 0.0190 | 1,463 | 7,838 | 109,732 | 0.0133 | 1,563 | 7,838 | 109,732 | 0.0142 |
| 10–19 | 864 | 10,177 | 142,478 | 0.0061 | 262 | 10,177 | 40,708 | 0.0064 | 830 | 10,177 | 142,478 | 0.0058 | 819 | 10,177 | 142,478 | 0.0057 |
| 20–29 | 560 | 9,572 | 134,008 | 0.0042 | 196 | 9,572 | 38,288 | 0.0051 | 591 | 9,572 | 134,008 | 0.0044 | 524 | 9,572 | 134,008 | 0.0039 |
| 30–39 | 942 | 11,302 | 158,228 | 0.0060 | 322 | 11,302 | 45,208 | 0.0071 | 939 | 11,302 | 158,228 | 0.0059 | 858 | 11,302 | 158,228 | 0.0054 |
| 40–49 | 1,319 | 15,843 | 221,802 | 0.0059 | 464 | 15,843 | 63,372 | 0.0073 | 1,321 | 15,843 | 221,802 | 0.0060 | 1,156 | 15,843 | 221,802 | 0.0052 |
| 50–59 | 1,798 | 15,835 | 221,690 | 0.0081 | 662 | 15,835 | 63,340 | 0.0105 | 1,915 | 15,835 | 221,690 | 0.0086 | 1,753 | 15,835 | 221,690 | 0.0079 |
| 60–69 | 1,971 | 11,446 | 160,244 | 0.0123 | 751 | 11,446 | 45,784 | 0.0164 | 2,196 | 11,446 | 160,244 | 0.0137 | 1,924 | 11,446 | 160,244 | 0.0120 |
| 70–79 | 1,718 | 7,466 | 104,524 | 0.0164 | 745 | 7,466 | 29,864 | 0.0249 | 1,866 | 7,466 | 104,524 | 0.0179 | 1,740 | 7,466 | 104,524 | 0.0166 |
| ≥80 | 730 | 2,929 | 41,006 | 0.0178 | 304 | 2,929 | 11,716 | 0.0259 | 816 | 2,929 | 41,006 | 0.0199 | 734 | 2,929 | 41,006 | 0.0179 |
| E |  |  |  |  |  |  |  |  |  |  |  |  |  |  |  |  |
| All ages | 609 | 92,814 | 1,299,396 | 0.0005 | 156 | 92,814 | 371,256 | 0.0004 | 511 | 92,814 | 1,299,396 | 0.0004 | 627 | 92,814 | 1,299,396 | 0.0005 |
| 0–9 | 72 | 7,838 | 109,732 | 0.0007 | 29 | 7,838 | 31,352 | 0.0009 | 72 | 7,838 | 109,732 | 0.0007 | 88 | 7,838 | 109,732 | 0.0008 |
| 10–19 | 21 | 10,177 | 142,478 | 0.0001 | 8 | 10,177 | 40,708 | 0.0002 | 32 | 10,177 | 142,478 | 0.0002 | 41 | 10,177 | 142,478 | 0.0003 |
| 20–29 | 29 | 9,572 | 134,008 | 0.0002 | 12 | 9,572 | 38,288 | 0.0003 | 27 | 9,572 | 134,008 | 0.0002 | 41 | 9,572 | 134,008 | 0.0003 |
| 30–39 | 47 | 11,302 | 158,228 | 0.0003 | 9 | 11,302 | 45,208 | 0.0002 | 42 | 11,302 | 158,228 | 0.0003 | 53 | 11,302 | 158,228 | 0.0003 |
| 40–49 | 57 | 15,843 | 221,802 | 0.0003 | 18 | 15,843 | 63,372 | 0.0003 | 55 | 15,843 | 221,802 | 0.0002 | 53 | 15,843 | 221,802 | 0.0002 |
| 50–59 | 103 | 15,835 | 221,690 | 0.0005 | 26 | 15,835 | 63,340 | 0.0004 | 92 | 15,835 | 221,690 | 0.0004 | 88 | 15,835 | 221,690 | 0.0004 |
| 60–69 | 109 | 11,446 | 160,244 | 0.0007 | 15 | 11,446 | 45,784 | 0.0003 | 73 | 11,446 | 160,244 | 0.0005 | 97 | 11,446 | 160,244 | 0.0006 |
| 70–79 | 104 | 7,466 | 104,524 | 0.0010 | 23 | 7,466 | 29,864 | 0.0008 | 71 | 7,466 | 104,524 | 0.0007 | 88 | 7,466 | 104,524 | 0.0008 |
| ≥80 | 67 | 2,929 | 41,006 | 0.0016 | 16 | 2,929 | 11,716 | 0.0014 | 47 | 2,929 | 41,006 | 0.0011 | 78 | 2,929 | 41,006 | 0.0019 |

H = hospitalization; O = outpatient clinic visit; E = emergency depart visit

Pop = total population of wildfire-exposed areas; PY = person-years; IR = incidence rate

Pre-wildfire (April 22, 2017, to May 5, 2017), wildfire (May 6, 2017, to May 9, 2017), immediate post-wildfire (May 10, 2017, to May 23, 2017), and extended post-wildfire (May 24, 2017, to June 6, 2017) periods

**Table S3.** Relative Risks of Disease-specific Respiratory Disease Healthcare Utilization among the Population of Wildfire-Exposed Areas by Age Groups

(a) Samcheok (Direct-exposure area)

| Variable | Age Group (year) | 2017 [W] RR (95% CI) | 2017 [IP] RR (95% CI) | 2017 [EP] RR (95% CI) | 2017/2018 [W] RRR (95% CI) | 2017/2018 [IP] RRR (95% CI) | 2017/2018 [EP] RRR (95% CI) |
| --- | --- | --- | --- | --- | --- | --- | --- |
| Pneumonia | All age | 1.79(1.66–1.93) | 0.75(0.73–0.78) | 1.20(1.14–1.26) | 1.16(1.03–1.30) | 0.85(0.79–0.91) | 1.37(1.26–1.51) |
|  | ≥ 20 | 1.11(1.02–1.21) | 0.69(0.67–0.71) | 1.08(1.03–1.14) | 0.89(0.78–1.02) | 0.90(0.84–0.97) | 1.37(1.25–1.51) |
|  | 0-9 | 2.48(2.28–2.70) | 0.78(0.76–0.81) | 1.39(1.31–1.47) | 1.12(0.98–1.28) | 0.60(0.56–0.65) | 1.21(1.09–1.34) |
|  | 10-19 | 0.53(0.50–0.55) | 1.38(1.35–1.42) | 1.78(1.69–1.86) | 0.40(0.36–0.45) | 0.88(0.82–0.95) | 1.14(1.02–1.28) |
|  | 20-65 | 1.11(1.02–1.21) | 0.87(0.84–0.90) | 1.37(1.30–1.44) | 1.80(1.58–2.05) | 2.17(2.01–2.33) | 3.52(3.21–3.86) |
|  | >65 | 1.09(1.00–1.18) | 0.60(0.58–0.62) | 0.98(0.93–1.03) | 0.93(0.82–1.05) | 0.79(0.74–0.85) | 1.10(1.02–1.19) |
| Acute bronchitis | All age | 1.92(1.77–2.08) | 0.78(0.76–0.81) | 1.28(1.21–1.34) | 1.35(1.21–1.52) | 0.99(0.92–1.05) | 1.80(1.65–1.96) |
|  | ≥ 20 | 1.97(1.82–2.15) | 0.78(0.75–0.80) | 1.25(1.18–1.31) | 1.54(1.37–1.74) | 1.06(0.99–1.13) | 1.87(1.72–2.04) |
|  | 0-9 | 2.21(2.04–2.38) | 0.83(0.80–0.86) | 1.44(1.37–1.52) | 1.36(1.21–1.52) | 0.93(0.86–1.00) | 1.82(1.65–1.99) |
|  | 10-19 | 1.31(1.21–1.42) | 0.70(0.67–0.72) | 0.95(0.90–1.00) | 1.65(1.48–1.85) | 1.25(1.17–1.34) | 1.92(1.77–2.09) |
|  | 20-65 | 2.06(1.91–2.23) | 0.80(0.78–0.83) | 1.20(1.14–1.26) | 1.56(1.40–1.74) | 1.00(0.94–1.06) | 1.75(1.62–1.90) |
|  | >65 | 1.89(1.72–2.08) | 0.72(0.69–0.75) | 1.27(1.20–1.34) | 1.19(1.04–1.37) | 0.93(0.86–1.01) | 1.72(1.56–1.90) |
| Acute bronchiolitis | All age | 1.88(1.73–2.04) | 0.85(0.81–0.88) | 1.44(1.35–1.52) | 0.88(0.78–1.00) | 0.92(0.85–1.00) | 1.68(1.51–1.86) |
|  | ≥ 20 | 7.19(6.54–7.90) | 0.75(0.73–0.78) | 0.74(0.70–0.78) | 1.76(1.52–2.04) | 0.36(0.34–0.39) | 0.37(0.33–0.41) |
|  | 0-9 | 1.76(1.62–1.91) | 0.96(0.92–0.99) | 1.92(1.80–2.03) | 0.72(0.63–0.82) | 0.98(0.94–1.03) | 2.03(1.87–2.21) |
|  | 10-19 |  |  |  |  |  |  |
|  | 20-65 | 3.31(2.98–3.67) | 0.47(0.45–0.48) | 0.35(0.33–0.37) | 3.26(2.94–3.62) | 0.60(0.55–0.65) | 0.42(0.38–0.47) |
|  | >65 | 0.73(0.64–0.83) | 2.93(2.80–3.07) | 5.59(5.19–6.02) | 1.75(1.52–2.00) | 3.33(3.15–3.52) | 4.58(4.19–5.00) |
| Unspecified lower respiratory tract infection | All age | 2.14(1.98–2.32) | 0.79(0.77–0.82) | 1.35(1.28–1.43) | 0.66(0.58–0.75) | 0.75(0.70–0.80) | 0.94(0.85–1.04) |
|  | ≥ 20 | 2.48(2.28–2.69) | 0.82(0.80–0.85) | 1.47(1.39–1.56) | 2.12(1.86–2.42) | 1.51(1.40–1.64) | 2.57(2.33–2.83) |
|  | 0-9 |  | 1.53(1.00–2.33) | 1.43(0.79–2.58) |  | 0.46(0.23–0.91) | 0.18(0.08–0.42) |
|  | 10-19 |  |  |  |  |  |  |
|  | 20-65 | 1.43(1.30–1.56) | 0.78(0.75–0.82) | 1.24(1.17–1.32) | 0.57(0.50–0.66) | 0.77(0.73–0.80) | 1.06(0.95–1.18) |
|  | >65 | 3.34(3.07–3.64) | 0.82(0.79–0.85) | 1.39(1.32–1.48) | 1.20(1.03–1.41) | 1.08(0.98–1.19) | 0.84(0.74–0.95) |
| COPD | All age | 1.71(1.57–1.87) | 0.76(0.74–0.79) | 1.30(1.23–1.37) | 1.16(1.02–1.32) | 0.92(0.86–0.99) | 1.73(1.58–1.90) |
|  | ≥ 20 | 1.73(1.58–1.89) | 0.75(0.73–0.78) | 1.27(1.20–1.34) | 1.30(1.15–1.47) | 0.99(0.93–1.06) | 1.78(1.63–1.94) |
|  | 20-65 | 1.83(1.67–2.00) | 0.79(0.76–0.81) | 1.19(1.13–1.26) | 1.17(1.04–1.33) | 0.82(0.77–0.86) | 1.55(1.41–1.70) |
|  | >65 | 1.64(1.50–1.79) | 0.72(0.70–0.75) | 1.34(1.27–1.41) | 1.20(1.05–1.38) | 1.01(0.94–1.09) | 1.88(1.70–2.07) |
| Asthma | All age | 1.69(1.56–1.84) | 0.78(0.76–0.81) | 1.33(1.27–1.41) | 1.05(0.93–1.18) | 0.94(0.88–1.01) | 1.69(1.54–1.84) |
|  | ≥ 20 | 1.72(1.58–1.88) | 0.76(0.74–0.79) | 1.29(1.23–1.36) | 1.40(1.24–1.58) | 1.11(1.04–1.19) | 2.06(1.89–2.24) |
|  | 0-9 | 2.26(2.07–2.46) | 0.84(0.81–0.87) | 1.52(1.43–1.61) | 1.30(1.15–1.47) | 0.95(0.88–1.02) | 1.77(1.60–1.95) |
|  | 10-19 | 0.61(0.57–0.66) | 0.71(0.69–0.73) | 1.02(0.97–1.07) | 0.69(0.61–0.77) | 1.13(1.06–1.21) | 1.56(1.45–1.67) |
|  | 20-65 | 1.73(1.60–1.88) | 0.78(0.76–0.81) | 1.23(1.17–1.30) | 1.32(1.17–1.48) | 1.03(0.97–1.10) | 1.87(1.72–2.04) |
|  | >65 | 1.81(1.65–1.99) | 0.75(0.72–0.77) | 1.41(1.34–1.50) | 0.81(0.71–0.94) | 0.79(0.74–0.85) | 1.43(1.35–1.51) |
| Bronchiectasis | All age | 1.68(1.52–1.85) | 0.57(0.55–0.60) | 1.03(0.96–1.10) | 0.88(0.83–0.94) | 0.33(0.29–0.38) | 0.40(0.37–0.44) |
|  | ≥ 20 | 1.68(1.52–1.85) | 0.57(0.55–0.60) | 1.03(0.96–1.10) | 0.56(0.52–0.60) | 0.38(0.32–0.45) | 0.45(0.41–0.50) |
|  | 0-9 |  |  |  |  |  |  |
|  | 10-19 |  |  |  |  |  |  |
|  | 20-65 | 4.20(3.87–4.57) | 0.96(0.93–1.00) | 1.57(1.47–1.67) | 1.37(1.28–1.45) | 0.86(0.74–0.99) | 0.76(0.69–0.83) |
|  | >65 | 0.97(0.86–1.09) | 0.69(0.66–0.73) | 0.84(0.78–0.91) | 1.04(0.98–1.11) | 0.10(0.09–0.11) | 0.32(0.29–0.35) |

Reference - Pre-wildfire period (April 22, 2017 - May 5, 2017), [W] = wildfire period (May 6, 2017 - May 9, 2017); [IP] = immediate post-wildfire period (May 10, 2017 - May 23, 2017); [EP] = extended post-wildfire period (May 24, 2017 - June 6, 2017)

RR – Risk ratio, RRR – Ratio of RR, CI – Confidence interval

Quasi-Poisson regression model with a log-link function to allow for over-dispersion to estimate the association of daily healthcare use with air pollution indicators, including PM, O_3_, NO_2_, CO, and SO_2_. The statistical model was adjusted for day of the weekend or holiday, wind direction, and relative humidity.

(b) Donghae (Indirect-exposure area)

| **Variable** | **Age Group (year)** | **2017 [W] RR (95% CI)** | **2017 [IP] RR (95% CI)** | **2017 [EP] RR (95% CI)** | **2017/2018 [W] RRR (95% CI)** | **2017/2018 [IP] RRR (95% CI)** | **2017/2018 [EP] RRR (95% CI)** |
| --- | --- | --- | --- | --- | --- | --- | --- |
| Pneumonia | All age | 1.23(1.16–1.29) | 0.60(0.58–0.62) | 1.09(1.04–1.13) | 0.50(0.47–0.54) | 0.41(0.39–0.43) | 0.74(0.69–0.78) |
|  | ≥ 20 | 1.70(1.61–1.79) | 0.54(0.53–0.56) | 0.85(0.81–0.88) | 0.85(0.79–0.92) | 0.41(0.39–0.43) | 0.58(0.55–0.62) |
|  | 0-9 | 0.80(0.76–0.86) | 0.70(0.67–0.72) | 1.56(1.49–1.64) | 0.26(0.24–0.29) | 0.46(0.43–0.49) | 1.09(1.01–1.18) |
|  | 10-19 | 1.13(1.05–1.22) | 0.70(0.67–0.74) | 0.58(0.54–0.61) | 0.98(0.89–1.08) | 0.57(0.53–0.62) | 0.43(0.40–0.47) |
|  | 20-65 | 1.12(1.06–1.18) | 0.58(0.56–0.60) | 0.86(0.82–0.89) | 0.78(0.72–0.84) | 0.58(0.56–0.60) | 0.89(0.84–0.94) |
|  | >65 | 1.87(1.76–1.99) | 0.56(0.54–0.58) | 0.89(0.85–0.93) | 0.80(0.74–0.87) | 0.33(0.31–0.35) | 0.49(0.46–0.52) |
| Acute bronchitis | All age | 1.03(0.97–1.08) | 0.66(0.64–0.68) | 1.18(1.13–1.23) | 0.46(0.44–0.49) | 0.50(0.47–0.52) | 0.84(0.79–0.89) |
|  | ≥ 20 | 1.18(1.12–1.25) | 0.64(0.62–0.66) | 1.09(1.05–1.14) | 0.51(0.47–0.55) | 0.47(0.45–0.49) | 0.74(0.70–0.79) |
|  | 0-9 | 0.70(0.66–0.74) | 0.71(0.69–0.74) | 1.58(1.52–1.66) | 0.30(0.28–0.33) | 0.55(0.52–0.58) | 1.10(1.04–1.17) |
|  | 10-19 | 1.14(1.09–1.20) | 0.66(0.64–0.68) | 0.94(0.90–0.98) | 0.77(0.72–0.83) | 0.59(0.56–0.61) | 0.89(0.84–0.94) |
|  | 20-65 | 1.13(1.07–1.19) | 0.67(0.65–0.69) | 1.06(1.02–1.11) | 0.54(0.50–0.58) | 0.51(0.49–0.53) | 0.79(0.75–0.83) |
|  | >65 | 1.35(1.26–1.44) | 0.58(0.56–0.60) | 1.13(1.07–1.18) | 0.49(0.45–0.53) | 0.40(0.38–0.43) | 0.66(0.62–0.71) |
| Acute bronchiolitis | All age | 1.07(1.00–1.14) | 0.75(0.72–0.78) | 1.49(1.42–1.57) | 0.46(0.42–0.51) | 0.52(0.49–0.55) | 1.10(1.03–1.19) |
|  | ≥ 20 | 1.37(1.28–1.47) | 0.73(0.70–0.76) | 1.30(1.23–1.37) | 0.83(0.76–0.91) | 0.90(0.84–0.96) | 1.28(1.22–1.36) |
|  | 0-9 | 0.83(0.77–0.89) | 0.74(0.71–0.77) | 1.43(1.35–1.51) | 0.34(0.30–0.37) | 0.42(0.40–0.45) | 0.80(0.74–0.87) |
|  | 10-19 | 4.03(1.95–8.33) | 2.87(1.18–7.00) | 0.38(0.18–0.79) | 4.05(1.96–8.38) | 2.87(1.18–7.01) | 0.20(0.09–0.41) |
|  | 20-65 | 1.31(1.21–1.41) | 0.78(0.74–0.81) | 1.41(1.33–1.49) | 0.49(0.44–0.54) | 0.54(0.51–0.58) | 0.87(0.81–0.93) |
|  | >65 | 0.92(0.84–1.00) | 0.90(0.85–0.95) | 1.13(1.05–1.22) | 0.88(0.80–0.97) | 0.83(0.78–0.89) | 1.65(1.51–1.80) |
| Unspecified lower respiratory tract infection | All age | 1.05(0.99–1.12) | 0.64(0.62–0.66) | 1.24(1.18–1.30) | 0.48(0.45–0.52) | 0.49(0.47–0.52) | 0.86(0.80–0.91) |
|  | ≥ 20 | 1.06(1.00–1.13) | 0.63(0.61–0.65) | 1.21(1.15–1.27) | 0.47(0.44–0.51) | 0.48(0.46–0.51) | 0.81(0.76–0.87) |
|  | 0-9 | 0.46(0.42–0.50) | 1.00(0.95–1.04) | 1.34(1.26–1.44) | 0.12(0.11–0.13) | 0.46(0.44–0.48) | 0.56(0.52–0.61) |
|  | 10-19 | 0.88(0.81–0.95) | 0.86(0.83–0.90) | 1.31(1.23–1.38) | 1.11(1.01–1.23) | 0.67(0.63–0.71) | 1.28(1.21–1.36) |
|  | 20-65 | 0.95(0.89–1.01) | 0.70(0.68–0.72) | 1.38(1.32–1.45) | 0.44(0.40–0.47) | 0.57(0.54–0.60) | 1.02(0.95–1.08) |
|  | >65 | 1.02(0.95–1.08) | 0.51(0.50–0.53) | 0.96(0.91–1.00) | 0.42(0.40–0.44) | 0.37(0.35–0.39) | 0.55(0.52–0.59) |
| COPD | All age | 1.15(1.08–1.21) | 0.65(0.62–0.67) | 1.11(1.06–1.16) | 0.46(0.43–0.50) | 0.49(0.47–0.52) | 0.75(0.70–0.80) |
|  | ≥ 20 | 1.25(1.18–1.33) | 0.63(0.61–0.65) | 1.06(1.01–1.11) | 0.51(0.47–0.55) | 0.48(0.46–0.51) | 0.72(0.68–0.76) |
|  | 20-65 | 1.15(1.09–1.22) | 0.62(0.60–0.64) | 0.95(0.91–0.99) | 0.50(0.46–0.54) | 0.49(0.46–0.51) | 0.69(0.65–0.74) |
|  | >65 | 1.40(1.31–1.49) | 0.64(0.62–0.67) | 1.18(1.12–1.24) | 0.50(0.46–0.54) | 0.47(0.44–0.49) | 0.69(0.65–0.74) |
| Asthma | All age | 1.01(0.95–1.07) | 0.63(0.61–0.65) | 1.22(1.16–1.27) | 0.44(0.42–0.47) | 0.46(0.44–0.48) | 0.81(0.76–0.86) |
|  | ≥ 20 | 1.21(1.14–1.29) | 0.59(0.57–0.61) | 1.03(0.99–1.08) | 0.58(0.53–0.63) | 0.43(0.41–0.46) | 0.76(0.72–0.81) |
|  | 0-9 | 0.59(0.55–0.62) | 0.73(0.71–0.76) | 1.97(1.88–2.07) | 0.22(0.21–0.24) | 0.55(0.52–0.58) | 1.36(1.27–1.45) |
|  | 10-19 | 1.25(1.17–1.35) | 0.83(0.80–0.87) | 1.14(1.08–1.21) | 0.88(0.80–0.96) | 0.65(0.61–0.69) | 0.83(0.77–0.89) |
|  | 20-65 | 1.15(1.08–1.21) | 0.62(0.60–0.64) | 1.08(1.03–1.13) | 0.63(0.58–0.68) | 0.43(0.41–0.45) | 0.76(0.71–0.81) |
|  | >65 | 1.37(1.29–1.47) | 0.55(0.53–0.57) | 0.95(0.91–1.00) | 0.44(0.41–0.48) | 0.38(0.36–0.41) | 0.55(0.51–0.59) |
| Bronchiectasis | All age | 1.34(1.26–1.42) | 0.83(0.80–0.85) | 1.60(1.54–1.67) | 0.57(0.53–0.61) | 0.47(0.45–0.50) | 0.80(0.75–0.85) |
|  | ≥ 20 | 1.34(1.26–1.42) | 0.83(0.80–0.85) | 1.60(1.54–1.67) | 1.02(0.94–1.12) | 0.79(0.75–0.83) | 1.80(1.69–1.92) |
|  | 0-9 |  |  |  |  |  |  |
|  | 10-19 |  |  |  |  |  |  |
|  | 20-65 | 1.89(1.77–2.02) | 1.20(1.15–1.24) | 1.53(1.46–1.62) | 2.12(1.93–2.33) | 0.80(0.75–0.84) | 1.04(0.96–1.11) |
|  | >65 | 0.68(0.62–0.74) | 0.77(0.74–0.80) | 2.01(1.89–2.14) | 0.22(0.20–0.24) | 0.51(0.48–0.54) | 0.95(0.88–1.02) |

Reference - pre-wildfire period (April 22, 2017 - May 5, 2017), [W] = wildfire period (May 6, 2017 - May 9, 2017); [IP] = immediate post-wildfire period (May 10, 2017 - May 23, 2017); [EP] = extended post-wildfire period (May 24, 2017 - June 6, 2017)

RR – Risk ratio, RRR – Ratio of RR, CI – Confidence interval

Quasi-Poisson regression model with a log-link function to allow for over-dispersion to estimate the association of daily health care use with air pollution indicators, including PM, O_3_, NO_2_, CO, and SO_2_. The statistical model was adjusted for the day of weekend or holiday, wind
